# Dynamic metabolomics differentiates between carbon and energy starvation in recombinant *Saccharomyces cerevisiae* fermenting xylose

**DOI:** 10.1186/1754-6834-5-34

**Published:** 2012-05-15

**Authors:** Basti Bergdahl, Dominik Heer, Uwe Sauer, Bärbel Hahn-Hägerdal, Ed WJ van Niel

**Affiliations:** 1 Applied Microbiology, Lund University, PO Box 124, SE-221 00, Lund, Sweden; 2 ETH Zurich, Zurich, 8093, Switzerland

**Keywords:** Metabolomics, Yeast metabolism, Xylose fermentation, Metabolic status, Starvation, Bioethanol

## Abstract

**Background:**

The concerted effects of changes in gene expression due to changes in the environment are ultimately reflected in the metabolome. Dynamics of metabolite concentrations under a certain condition can therefore give a description of the cellular state with a high degree of functional information. We used this potential to evaluate the metabolic status of two recombinant strains of *Saccharomyces cerevisiae* during anaerobic batch fermentation of a glucose/xylose mixture. Two isogenic strains were studied, differing only in the pathways used for xylose assimilation: the oxidoreductive pathway with xylose reductase (XR) and xylitol dehydrogenase (XDH) or the isomerization pathway with xylose isomerase (XI). The isogenic relationship between the two strains ascertains that the observed responses are a result of the particular xylose pathway and not due to unknown changes in regulatory systems. An increased understanding of the physiological state of these strains is important for further development of efficient pentose-utilizing strains for bioethanol production.

**Results:**

Using LC-MS/MS we determined the dynamics in the concentrations of intracellular metabolites in central carbon metabolism, nine amino acids, the purine nucleotides and redox cofactors. The general response to the transition from glucose to xylose was increased concentrations of amino acids and TCA-cycle intermediates, and decreased concentrations of sugar phosphates and redox cofactors. The two strains investigated had significantly different uptake rates of xylose which led to an enhanced response in the XI-strain. Despite the difference in xylose uptake rate, the adenylate energy charge remained high and stable around 0.8 in both strains. In contrast to the adenylate pool, large changes were observed in the guanylate pool.

**Conclusions:**

The low uptake of xylose by the XI-strain led to several distinguished responses: depletion of key metabolites in glycolysis and NADPH, a reduced GTP/GDP ratio and accumulation of PEP and aromatic amino acids. These changes are strong indicators of carbon starvation. The XR/XDH-strain displayed few such traits. The coexistence of these traits and a stable adenylate charge indicates that xylose supplies energy to the cells but does not suppress a response similar to carbon starvation. Particular signals may play a role in the latter, of which the GTP/GMP ratio could be a candidate as it decreased significantly in both strains.

## Background

The yeast *Saccharomyces cerevisiae* has been the organism of choice in the food and beverage industry due to its excellent growth and fermentation capabilities under anaerobic conditions. These characteristics combined with high tolerance to low pH and high sugar and ethanol concentrations yields a production organism, which is very robust in industrial processes. Thus, *S. cerevisiae* has also become the preferred organism for the production of biofuels and fine chemicals [1]. An important step towards the replacement of fossil raw materials is the efficient utilization of renewable lignocellulose. This raw material is generated in the forest and agricultural sectors, and contains carbohydrates which can be converted into fuel-grade ethanol by fermentation [2]. The pentose sugar xylose constitutes a major fraction of the sugar monomers obtained after hydrolysis of certain lignocellulose materials [3,4] and complete utilization of xylose is therefore necessary to obtain competitive process economics [5].

**Figure 1:**
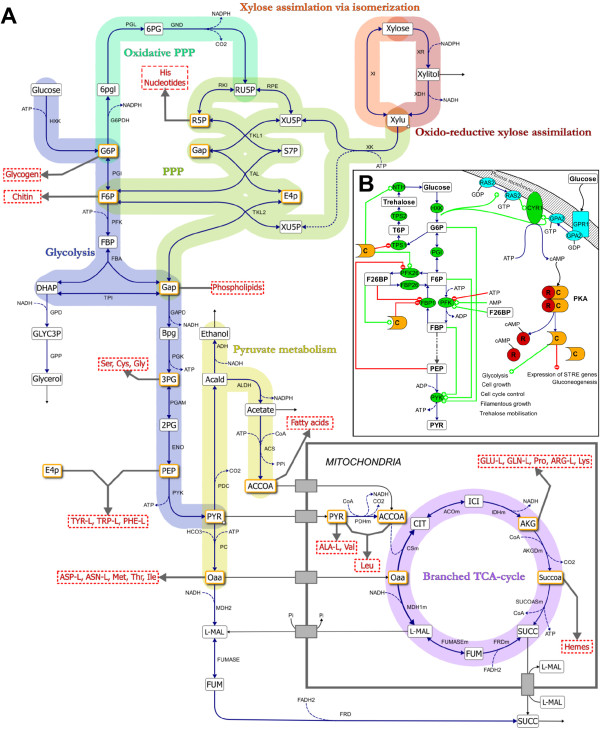
**Overview of the main metabolic reactions in central carbon metabolism.A**) Xylose can be assimilated through two pathways: the fungal oxidoreductive pathway consisting of XR and XDH or the bacterial isomerization pathway via an XI. The xylulose formed by the two pathways is phosphorylated by XK and channelled through the non-oxidative PPP yielding 2/3 moles F6P and 1/3 moles GAP per mole substrate. These metabolites enter glycolysis to generate ATP in the conversion of PEP to pyruvate and the oxidative PPP to regenerate NADPH. Pyruvate is converted into ethanol, acetate and oxaloacetate, which is transported into the mitochondria for amino acid synthesis. Under anaerobic conditions succinate dehydrogenase is not operational which results in a branched TCA-cycle with succinate as the end product. The twelve precursor metabolites which are required for biosynthesis of macromolecules are highlighted in orange colour. Metabolites in capital letters have been measured in the current study. **B**) Glycolysis is activated through the induction of the Ras/cAMP/PKA pathway. cAMP formed by adenylate cyclase (CYR1) interacts with the regulatory subunits (R) of PKA which releases the catalytic subunits (C). The active PKA phosphorylates PFK26 which subsequently produces F26BP from F6P and ATP. F26BP is an essential activator of PFK1 which phosphorylates F6P to FBP. The production of FBP in turn activates PYK and ATP generation. Production of ATP and ethanol depends on a high catalytic activity of PYK which requires the simultaneous presence of G6P, F6P and FBP. PKA and F26BP also efficiently inactivate gluconeogenesis by inhibiting FBP1.

*S. cerevisiae* cannot naturally utilize xylose and has therefore been extensively engineered to acquire this trait as summarized in several recent reviews [6-9]. To enable xylose utilization and fermentation to ethanol by *S. cerevisiae*, one of two heterologous pathways have been introduced: the oxido-reductive pathway or the isomerisation pathway [10] (Figure 1A). The oxido-reductive pathway is found in fungi and consists of two enzymes, a NAD(P)H-dependent xylose reductase (XR) and a NAD-dependent xylitol dehydrogenase (XDH). Some XR enzymes can use both NADH and NADPH as cofactor although with a preference for the latter [11]. This causes a cofactor imbalance between the XR and XDH reactions and leads to xylitol formation and reduced ethanol yields [12-14]. This does not occur in the isomerisation pathway, which only consists of a cofactor-independent xylose isomerase (XI). The XI with highest activity when expressed in *S. cerevisiae* originates from the fungus *Piromyces sp*. Even so, the activity of this XI is too low to enable anaerobic growth on xylose without further evolution or adaptation of the recombinant strain [10,15].

The rational design of industrially applicable microorganisms requires an understanding of the cellular processes that give rise to a certain phenotype. The functional information about a cellular phenotype is found in the biochemical processes that occur in response to environmental conditions and is commonly viewed as the connection between the three major ‘omes in the cell: transcriptome, proteome and metabolome [16,17]. Both proteins and metabolites are directly involved in the cellular biochemistry and thereby closely dictate the function of the organism [18]. The functional role of intracellular metabolites is eminent as they can influence the conversion rate by an enzyme, either as substrate, product or allosteric effector, act as signalling molecules and even influence the direction of enzymatic reactions through their thermodynamic properties [19]. The concerted effect of changes in gene expression in response to environmental variation is thus ultimately reflected in the metabolome. The dynamic changes in metabolite concentrations under a certain perturbation can therefore provide a description of the phenotype and the cellular state with a higher degree of functional information than a snap-shot of either the transcriptome or the proteome. We used this potential to evaluate the metabolic status of two isogenic recombinant strains of *S. cerevisiae* harbouring either the XR/XDH or the XI pathway during anaerobic batch fermentation of a glucose/xylose mixture. A recently developed method for quantification of intracellular concentrations of metabolites by LC-MS/MS [20] was used to determine the dynamic response of targeted metabolites in these recombinant strains when the carbon and energy source changed from glucose to xylose. The difference in xylose uptake rate between the XI-strain and the XR/XDH-strain allows us to propose responses in metabolite concentrations expected to occur during starvation for carbon and energy. The results suggest that xylose is primarily used as an energy source as both strains maintained a high energy charge during the transition to xylose fermentation while varying signs of carbon starvation were observed.

## Results

In this study we determined the dynamics in the concentrations of metabolites in glycolysis, the pentose phosphate pathway (PPP), the TCA cycle, nine amino acids, the purine nucleotides and redox cofactors, in two recombinant strains of *Saccharomyces cerevisiae*. The two strains have identical genetic background but employ two different pathways for xylose utilization; strain TMB 3057 [21] has the oxido-reductive fungal pathway consisting of XR and XDH from *Pichia stipitis* and strain TMB 3359 [22] has the isomerisation pathway consisting of the XI from *Piromyces sp*. Samples for determination of metabolite concentrations were collected at specific time points during anaerobic batch fermentation with 20 g/L glucose and 40 g/L xylose as substrates. Additionally, for the XR/XDH-strain measured intracellular metabolite concentrations were validated by evaluating the simultaneous thermodynamic feasibility of 237 metabolic and transport reactions at each sampling point.

### Fermentation performance

**Figure 2:**
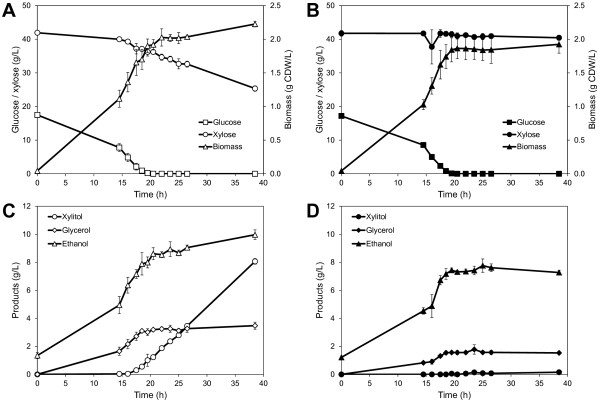
**Anaerobic batch fermentation profiles.** Fermentation profiles of substrate consumption, biomass and product formation of TMB3057 (XR/XDH) (**A** and **C**) and TMB3359 (XI) (**B** and **D**) Fermentation was performed in 2× YNB medium containing 20 g/L glucose and 40 g/L xylose. Figures show mean values of duplicate experiments and errors are given as the standard deviation of the mean (n = 4).

Both the XR/XDH-strain and the XI-strain consumed all glucose within 20.5 hours (Figure 2A and B) and in the same time the XR/XDH-strain co-consumed 5.7 ± 0.5 g/L xylose (Figure 2A), whereas the XI-strain only co-consumed 0.6 ± 0.2 g/L xylose (Figure 2B). Until this point the biomass yield per gram sugar consumed was similar for the two strains but higher yield of the by-products glycerol and xylitol were obtained with the XR/XDH-strain (Additional file 1: Table S1). After glucose depletion, the XR/XDH-strain consumed an additional 10.8 ± 0.1 g/L xylose (*r*_*xyl*_ = 0.30 g/g CDW/h) most of which was converted to xylitol and ethanol with a minor amount used for biomass and glycerol (Figure 2A and C). The XI-strain only consumed an additional 0.6 ± 0.1 g/L xylose (*r*_*xyl*_ = 0.036 g/g CDW/h) after glucose depletion (Figure 2B). During the xylose phase the XI-strain produced negligible amounts of xylitol (0.12 ± 0.02 g/L) in contrast to the XR/XDH-strain (6.8 ± 0.01 g/L xylitol) (Figure 2C and D).

### Validation of measured metabolite concentrations in the XR/XDH-strain

The metabolic response to a change in carbon source from glucose to xylose was determined by measuring intracellular metabolites at 11 time points during 24 hours of anaerobic batch cultivation. The first samples were collected at mid-exponential growth on glucose while the majority of samples were collected during the transition towards glucose depletion. The last samples were collected 18 hours after glucose exhaustion and represent a state when xylose is the sole carbon and energy source. Quadruplicate samples were collected at each time point and experiments were performed in duplicate for each strain. The final data set consisted of 185 samples and 44 measured metabolites.

The thermodynamic feasibility of 38 out of the 44 metabolite ranges measured in the XR/XDH-strain were validated using anNET toolbox [23]. These 38 metabolites were quantified using external standards. The metabolites that could not be quantified, due to lack of standards, were sedoheptulose 7-phosphate (S7P), acetyl-CoA (ACCOA), reduced and oxidized glutathione (GTTred and GTTox), FAD and FMN. All metabolites evaluated, apart from 1,3-bisphosphoglycerate (BPG), glucose 1-phosphate (G1P) and glyceraldehyde 3-phosphate (GAP), were within the feasible ranges in all 11 time points (see Additional file 2 for further details). This means that the measurements of the remaining 35 metabolites are of good quality as they allowed 237 reactions to be thermodynamically feasible simultaneously.

### Global analysis of the metabolite responses

**Figure 3:**
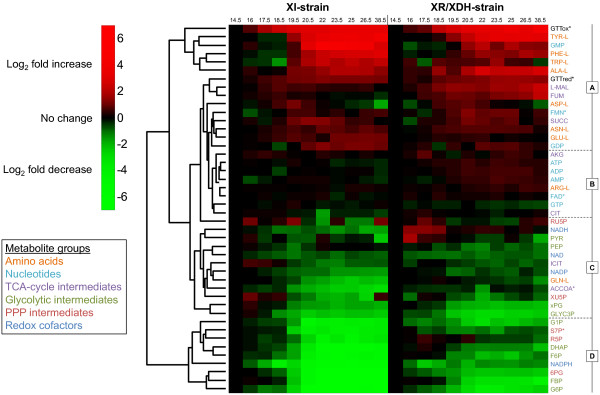
**Hierarchical clustering of time course profiles during fermentation of glucose and xylose.** The heat map was generated using either quantified metabolite concentrations or normalized areas of the chromatographic peaks (indicated by *) and shows the log_2_ fold change relative to the first sampling point (t = 14.5 h) which represents exponential growth on a mixture of glucose and xylose. The hierarchical clustering of metabolite profiles yields four major clusters: Cluster A contains 15 metabolites which increase in either of the two strains; Cluster B contains 8 metabolites which show little change during the fermentation; Cluster C contains 12 metabolites which decrease moderately in at least one strain; Cluster D contains 9 metabolites which decrease markedly in both the XR/XDH-strain and the XI-strain.

Hierarchical clustering of the metabolite data was used to obtain a global overview of the metabolomic response. To avoid performing this and subsequent analyses on metabolite data that might be erroneous and not physiologically relevant, GAP and BPG were removed from the data set (see Additional file 2). The clustering revealed four major groups of metabolites: one group where concentrations increased over time (Group A), one group where few changes occurred (Group B) and two groups where concentrations decreased over time (Group C and D) (Figure 3). In general, the response in metabolite concentrations was the same in both strains. Nearly all the measured amino acids are found in Group A. Specifically, all the aromatic amino acids clearly cluster in the top of this group. The nucleotide profiles displayed few changes and are found in Group B. The exceptions to this pattern were the guanine nucleotides GMP and GDP, which increased markedly in both strains during the fermentation, and GTP which decreased slightly. The redox cofactors NAD, NADH and NADP decreased moderately and are found in Group C. The concentration of NADPH, on the other hand, decreased significantly and is thus designated to Group D. Group D is otherwise dominated by sugar phosphates from both glycolysis and the PPP. The heat map also shows that the greatest shifts in the metabolite profiles were initiated immediately prior to glucose depletion.

### Identification of key metabolites and phases during batch fermentation

Principal component analysis (PCA) was used to determine the natural grouping of the sampling points based on quantified and validated concentrations, and to identify which metabolites have the highest influence on the separation of the data. The concentrations of pyruvate were not included in this analysis due to large errors in the measurements from the XI-strain (Additional file 1: Figure S6E). The first and second principal components (PC1 and PC2, respectively) accounted for 76.2% and 10.4% of the variance in the data, respectively (Additional file 1: Figure S1). The data points were separated by sampling time in PC1 and by strain background in PC2. The grouping of the data closely reflects the experimental structure. Samples from the glucose-xylose phase cluster together, illustrating similar metabolite profiles in the two strains during glucose metabolism, which reflects the isogenicity of the strains with the only difference being the xylose pathway. This difference becomes evident when glucose is depleted. Samples collected from the xylose phase clearly form two separate clusters, one for each strain. The grouping of the data from the XI-strain also reveals that there are three distinct phases during the fermentation: a glucose phase between 14.5 h and 18.5 h, a transition phase between 18.5 h and 22 h and a xylose phase between 22 h and 38.5 h. Among the 20 most influential metabolites on PC1 and PC2 the distribution was as follows: nine amino acids, five sugar phosphates, three carboxylic acids, two nucleotides and one redox cofactor (Additional file 1: Figure S1). This distribution agrees well with the results of the cluster analysis (Figure 3). It is worth noting that several parts of metabolism are represented by these metabolites and most importantly that the adenine nucleotides are not among them.

A two-sided t-test was used to establish whether the average concentration of each quantified metabolite was different between the two strains at each sampling point. This analysis showed that the number of metabolites that were significantly different between the strains (at a significance level of α = 0.05) increased from between 16 and 23 in the mixed sugar phase to between 29 and 33 in the xylose phase (Additional file 1: Table S2), which confirms the general pattern of the PCA analysis. The majority of the quantified metabolites (81%) were significantly different in more than half of the time points, including all the metabolites in Figure S1 (Additional file 1). The remaining metabolites that showed few differences between the two strains were the nucleotides and intermediates of the TCA cycle which are in Group B of the cluster analysis (Figure 3).

### **Dynamic changes in sugar phosphates, precursor metabolites and amino acids**

**Figure 4:**
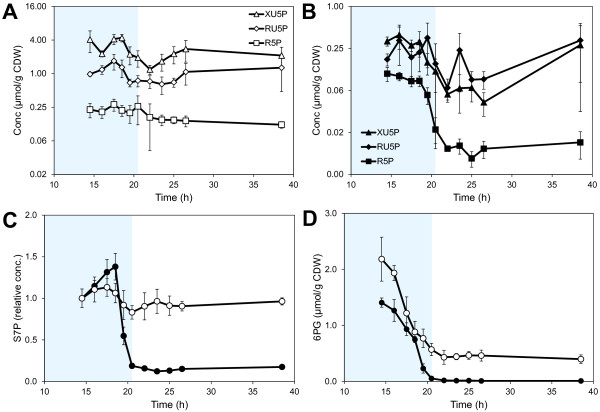
**Dynamics of metabolites in the pentose phosphate pathway.** Intracellular concentrations of **A**) XU5P, RU5P and R5P in TMB3057 (XR/XDH), **B**) XU5P, RU5P and R5P in TMB3359 (XI), **C**) S7P and **D**) 6PG during anaerobic batch fermentation of 20 g/L glucose and 40 g/L xylose using strains TMB3057 (XR/XDH, white markers) and TMB3359 (XI, black markers). Errors are given as 95% confidence intervals of the means calculated from duplicate experiments for each strain (5 ≤ n ≤ 10). The Y-axis in A and B is in log_2_ scale. The levels of S7P are calculated relative to the first time point at 14.5 h. The shaded area indicates the period until glucose depletion.

**Figure 5:**
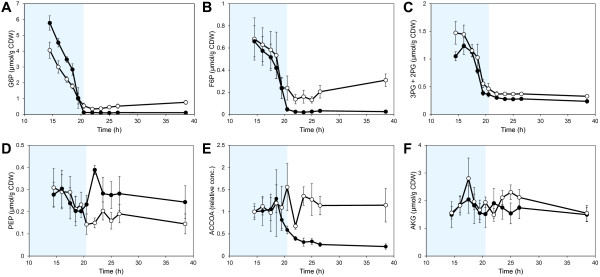
**Dynamics of important precursor metabolites.** Intracellular concentrations of **A**) G6P, **B**) F6P, **C**) the combined pool of 2PG and 3PG, **D**) PEP, **E**) ACCOA and **F**) AKG during anaerobic batch fermentation of 20 g/L glucose and 40 g/L xylose using strains TMB3057 (XR/XDH, white markers) and TMB3359 (XI, black markers). Errors are given as 95% confidence intervals of the means calculated from duplicate experiments for each strain (4 ≤ n ≤ 10). The shaded area indicates the period until glucose depletion. The levels of ACCOA are calculated relative to the first time point at 14.5 h. The shaded area indicates the period until glucose depletion.

The concentrations of sugar phosphates and amino acids showed the largest changes in concentration during the fermentation (Figure 3) and also had a high influence on the separation of the collected data (Additional file 1: Figure S1). Xylulose 5-phosphate (XU5P), ribulose 5-phosphate (RU5P) and ribose 5-phosphate (R5P) had a high influence on the separation of the two strains in the PCA analysis and the concentrations were also consistently lower in the XI-strain compared to the XR/XDH-strain (Figure 4A and B). The concentrations of these metabolites were rather stable in the XR/XDH-strain during the fermentation whereas the concentrations decreased in the XI-strain during the transition phase. The largest change was observed in the concentration of R5P which decreased by 9-fold (Figure 4B). The same change was observed in the level of S7P (Figure 4C). During the same period the level of S7P decreased only slightly (1.2-fold) in the XR/XDH-strain. S7P was not included in the PCA analysis due to the lack of quantitative data but the relative change during the transition phase could be calculated from the chromatographic peak areas. 6-phosphogluconate (6PG), the only metabolite measured in the oxidative part of the PPP, decreased significantly in both strains during the glucose- and transition phases (Figure 4D). The concentration decreased 5-fold in the XR/XDH-strain and nearly 75-fold in the XI-strain, leading to an almost depleted pool of 6PG in the latter. In addition to R5P several precursor metabolites in glycolysis and TCA-cycle also decreased during the transition from glucose to xylose fermentation (Figure 5). In the XR/XDH-strain the most significant changes occurred in the concentrations of glucose 6-phosphate (G6P), fructose 6-phosphate (F6P) and the combined pool of 2-phosphoglycerate (2PG) and 3-phosphoglycerate (3PG) which decreased by 8-, 3- and 4-fold, respectively (Figure 5 A-C). In the XI-strain the xPG pool also decreased 4-fold, but the decrease in hexose phosphates was more severe with 40- and 33-fold changes in the concentrations of G6P and F6P, respectively, resulting in near depletion of these pools after glucose exhaustion. In both strains the concentration of fructose 1,6-bisphosphate (FBP) decreased the most with 10- and 88-fold changes in the XR/XDH- and XI-strain, respectively (Additional file 1: Figure S5B). The concentrations of the metabolites in glycolysis generally decreased in both strains and the time course profiles correlated with decreasing concentration of extracellular glucose (Figure 3; Additional file 1: Figure S5). The exception to this pattern was phosphoenolpyruvate (PEP) which transiently increased 2-fold in concentration in the XI-strain after glucose depletion and then returned to the original concentration during the xylose phase (Figure 5D). The dynamic change in the level of ACCOA in the XI-strain followed that of the glycolytic intermediates and decreased by 3.7-fold (Figure 5E). In the XR/XDH-strain on the other hand there were few changes in the ACCOA concentration during the three phases. The concentration of α-ketoglutarate (AKG) varied notably in both strains during the three phases but within a rather small range from 1.5 μmol/g CDW to 2.3 μmol/g CDW on average (Figure 5F).

All the free amino acids measured in the current study, except glutamine (GLN-L), accumulated during the glucose and/or the transition phase (Figure 3; Additional file 1: Figure S7). The largest increase was seen in the pools of the aromatic amino acids phenylalanine (PHE-L), tryptophan (TRP-L) and tyrosine (TYR-L). The response was stronger in the XI-strain where the concentrations of PHE-L, TRP-L and TYR-L increased by 9-, 6- and 17-fold, respectively, while in the XR/XDH-strain the response was more moderate with 4-, 2- and 11-fold increases, respectively (Additional file 1: Figure S2). Concentrations of L-malate and fumarate also increased during the glucose and transition phases by ca. 2.3-fold and 1.4-fold in the XR/XDH-strain and XI-strain, respectively (Additional file 1: Figure S6 C and D). However, in the xylose phase these two carboxylic acids decreased in concentration in the XI-strain, whereas they continued to accumulate in the XR/XDH-strain, reaching around 5-fold higher concentrations compared with the glucose phase.

### **Dynamic changes in redox cofactors and nucleotides**

**Figure 6:**
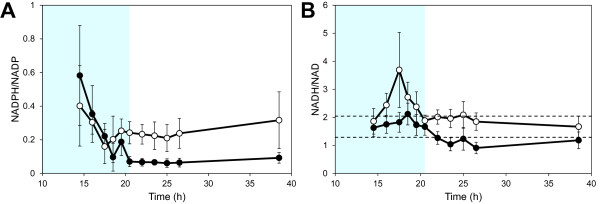
**Dynamics in redox cofactor ratios.** Dynamics in the NADPH/NADP (**A**) and NADH/NAD (**B**) ratios during anaerobic batch fermentation of 20 g/L glucose and 40 g/L xylose using strains TMB3057 (XR/XDH, white markers) and TMB3359 (XI, black markers). Dotted lines in B) represent the 95% confidence interval of the value in TMB3057 at time point 38.5 h. Errors are given as 95% confidence intervals of the means calculated from duplicate experiments for each strain (4 ≤ n ≤ 10). The shaded area indicates the period until glucose depletion.

In contrast to the consistent responses observed within the sugar phosphate and amino acid metabolite groups, the responses in redox cofactors and nucleotides were more diverse. The concentration of redox cofactors decreased similarly in both strains during the glucose- and transition phases, after which they levelled out in the xylose phase (Additional file 1: Figure S3). NADP was the only redox cofactor that appeared among the 20 most influential metabolites in the PCA analysis (Additional file 1: Figure S1) and the concentration was consistently higher in the XR/XDH-strain compared to the XI strain with a difference as high as 3.4-fold in the xylose phase (Additional file 1: Figure S3A). This large difference was due to a 2-fold reduction in concentration in the XI-strain whereas the concentration only changed by 27% in the XR/XDH-strain. During most of the glucose phase the concentration of NADPH was the same in both strains but in the xylose phase the concentration had decreased by 2.8-fold in the XR/XDH-strains and by nearly 22-fold in the XI-strain (Additional file 1: Figure S3B). As with several sugar phosphates this decrease led to a near depletion of the NADPH pool in the XI-strain. This depletion resulted in a 9-fold reduction of the NADPH/NADP ratio from 0.6 to ca. 0.07 (Figure 6A). In the XR/XDH-strain, on the other hand, few significant changes were observed in the NADPH/NADP ratio and the average ratio over all phases was 0.25 ± 0.03 (mean ± 95% CI, n = 79). In contrast to NADP, the concentration of NAD was on average 26% lower in the XR/XDH-strain compared to the XI-strain during all three phases and the change during the first two phases was almost 2-fold in both strains (Additional file 1: Figure S3C). There were few significant differences in NADH concentration between the XR/XDH-strain and the XI-strain (Additional file 1: Table S2) and the average concentration in the two strains during all three phases was 2.10 ± 0.20 μmol/g CDW (mean ± 95% CI, n = 85) and 1.91 ± 0.18 μmol/CDW (mean ± 95% CI, n = 85), respectively. This high consistency between the two strains in NAD(H) cofactors led to very few significant differences in the NADH/NAD ratio when comparing time point by time point (Figure 6B). However, there was a significant difference in the average ratio during the xylose phase which was 1.93 ± 0.15 (mean ± 95% CI, n = 41) in the XR/XDH-strain and 1.14 ± 0.11 (mean ± 95% CI, n = 39) in the XI-strain.

**Figure 7:**
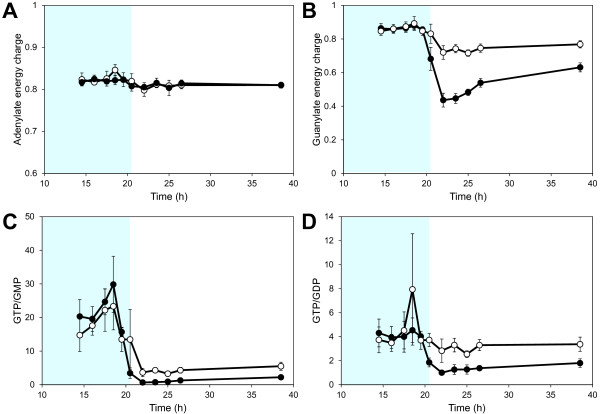
**Dynamics in different purine nucleotide ratios.** Dynamics in **A**) the adenylate energy charge, **B**) the guanylate energy charge, **C**) the GTP/GMP ratio and **D**) the GTP/GDP ratio during anaerobic batch fermentation of 20 g/L glucose and 40 g/L xylose using strains TMB3057 (XR/XDH, white markers) and TMB3359 (XI, black markers). The adenylate energy charge and the guanylate energy charge were calculated according to (ATP + ½ADP)/(ATP + ADP + AMP) and (GTP + ½GDP)/(GTP + GDP + GMP) , respectively. Errors are given as 95% confidence intervals of the means calculated from duplicate experiments for each strain (5 ≤ n ≤ 11). The shaded area indicates the period until glucose depletion.

The dynamic response of the adenine nucleotides was the same in both the XR/XDH-strain and the XI-strain. Furthermore, we found very few significant differences in the concentrations between the two strains (Additional file 1: Figure S4 A and B; Table S2). This was also reflected in the adenylate energy charge (AEC) which was nearly identical in both strains and remained at 0.82 during exponential growth on glucose and xylose fermentation (Figure 7A). This level of the AEC agrees very well with previously reported values for *S. cerevisiae* growing on glucose [36-38]. In contrast to adenylate nucleotides, the concentrations of the guanine nucleotides GMP and GDP changed significantly in the transition phase (Additional file 1: Figure S4C). In the XI-strain the concentrations increased 32- and 2.6-fold, respectively. In the XR/XDH-strain the concentrations also increased but to a lesser extent, i.e. 5- and 1.6-fold of GMP and GDP, respectively. The concentration of GTP decreased in the XR/XDH-strain throughout the three experimental phases, whereas in the XI-strain it gradually returned to a level close to the initial concentration (Additional file 1: Figure S4D). During the glucose phase both strains had nearly identical values of the guanylate energy charge (GEC) around 0.86, but during the transition phase the GEC dropped by almost 50% in the XI-strain to 0.44 whereas it stabilized at 0.72 in the XR/XDH-strain (Figure 7B). During the xylose phase the GEC recovered to 0.63 and 0.77 in the XI-strain and XR/XDH-strain, respectively. In addition to a drop in the GEC, the changes in GMP and GDP concentrations also resulted in altered GTP/GDP and GTP/GMP ratios. The dynamic response in the GTP/GMP ratio was similar in the two strains with decreases of 3- and 9-fold in the XR/XDH-strain and XI-strain, respectively (Figure 7C). The response in the GTP/GDP ratio was on the other hand different in the two strains (Figure 7D). The average GTP/GDP ratio was around 3.70 ± 0.34 (mean ± 95% CI, n = 81) in the XR/XDH-strain throughout the three experimental phases whereas it dropped from around 4.2 to ca 1.4 in the XI-strain.

## **Discussion**

For a culture of microorganisms to obtain balanced growth a careful coordination between nutrient assimilation, transformation of energy to generate ATP, redox balancing, biosynthesis and progression through the cell division cycle is required. In this study we determined the time-dependent changes of targeted metabolites in two recombinant strains of *S. cerevisiae*, harbouring either the XR/XDH- or the XI-pathway. This data allows us to evaluate the status of the five cellular processes required for balanced growth, from a metabolic perspective, when the cells shift from glucose to xylose fermentation.

### **Carbon assimilation is not the main limitation of growth on xylose**

When the carbon source changed from glucose to xylose there was a significant decrease in sugar uptake rate (Figure 2). Both strains had the same specific uptake rate of glucose, but that of xylose was 60% and 94% lower in the XR/XDH-strain and the XI-strain, respectively. These results agree well with previously reported performance data for both the XR/XDH-strain [39] and the XI-strain [22]. The results for the XI-strain also compare well with data from a related strain with the same genetic background, but with a different promoter controlling the expression of the XI-encoding gene [10]. The XI-strain used in the current study does not perform as well as other yeast strains harbouring an XI but which have also undergone extensive evolutionary engineering [40]. However, for the current investigation rationally designed strains were deliberately chosen to ensure isogenicity, except for the two initial xylose assimilating pathways, so that it can be reasonably assumed that the metabolism of the strains is controlled by the same regulatory system. The isogenic relationship between the two strains thus ensures that the observed responses are primarily a consequence of the metabolic impact of the adopted xylose assimilation pathway. When xylose is the sole carbon source mainly the high affinity glucose transporters are expressed [41] which have a K_m_ ≈ 130 mM for xylose [42]. Since the concentration of xylose during our experiments was on average double the K_m_ the transporters would be operating at 2/3 of the maximum velocity, which corresponds to approximately 0.26 g/g CDW/h (assuming a single transporter with V_max_ = 110 nmol/min/mg protein [42] and a cellular protein content of 40%). This value is probably an underestimation since more than one transporter is actually present, but the magnitude can be considered reasonable as it is close to the measured uptake rate in the XR/XDH-strain (0.3 g/g CDW/h). In the XI-strain on the other hand the measured uptake rate is several folds lower than the potential capacity, indicating that other factors downstream sugar transport is limiting the uptake of xylose. From this calculation it is difficult to evaluate whether transport is limiting in the XR/XDH-strain, but even though the uptake rate is 10-fold higher than in the XI-strain it is not able to grow on xylose. Attempts to improve the uptake of xylose in other XR/XDH-strains by introducing a heterologous glucose/xylose facilitator only had an effect at low xylose concentrations and the increased uptake did not improve biomass or ethanol yield from xylose [43]. This suggests that also in the XR/XDH-strain there are other limitations in addition to sugar uptake.

### **A carbon starvation response is displayed in biosynthetic metabolites**

In this study we measured 16 metabolites that are related to biosynthesis. Seven of these were precursor metabolites [44] required for biosynthesis of macromolecular building blocks (i.e. G6P, F6P, R5P, PEP, 3PG, AKG and ACCOA) (Figure 4; Figure 5) and the remaining nine were amino acids (Figure 3; Additional file 1: Figure S2 and S7). In total there are twelve essential precursor metabolites which are all produced in glycolysis, PPP and the TCA-cycle (Figure 1A). Hence, the rate of their formation is closely linked to the carbon uptake rate.

#### **Concentrations of PPP intermediates reflect the xylose uptake rate**

The measured concentrations of the metabolites in the non-oxidative PPP were markedly higher in the XR/XDH-strain compared to the XI-strain, even during growth on glucose (Figure 4A and B). This is most likely caused by the difference in xylose uptake. Between 14.5 h and 18.5 h the specific glucose uptake rate differed by less than 9% between the two strains (data not shown), however, when the uptake of xylose is also taken into account the difference in total sugar uptake rate is 45% (data not shown). The small difference with regard to glucose is consistent with the isogenic relationship between the two strains. Also the large difference in total sugar uptake rate, caused by a higher uptake of xylose by the XR/XDH-strain, agrees well with the higher concentrations of pentose sugar phosphates measured in this strain. The carbon channelled through the PPP during fermentative growth of natural *S. cerevisiae* is only around 15% of the glucose utilized [45] and the PEP generated by employing the PPP has been estimated to be less than 4% [46,47]. The role of PPP has thus been suggested as exclusively anabolic and not catabolic [47]. Native *S. cerevisiae* accumulates intracellular S7P during xylulose fermentation which was interpreted as insufficient activity of subsequent PPP enzymes [48]. This hypothesis seemed to be confirmed when overexpression of the four genes of the non-oxidative PPP led to increased fermentation of xylulose [49] and xylose [21]. Many recombinant xylose fermenting strains of *S. cerevisiae*, like the two strains used in the current investigation, currently carry this trait [50]. Nevertheless, recent metabolomics studies continue to point out overexpression of the non-oxidative PPP enzymes as targets for improved pentose utilization [37,38]. Based on the dynamics of the PPP intermediates in the current investigation with two isogenic strains, the limitation rather seems to have been shifted toward the initial xylose assimilating steps. This appears to be the case with the XI pathway in particular. One consequence of this limitation is that the XI-strain is unable to sustain a flux towards 6PG formation (Figure 4D). 6PG is formed and consumed in two irreversible reactions catalysed by 6-phosphogluconolactonase (PGL; EC 3.1.1.31) and 6-phosphogluconate dehydrogenase (GND; EC 1.1.1.44), respectively (Figure 1A). Hence, the concentration of 6PG depends on the ratio between the rates of formation and consumption. The depletion of 6PG in the XI-strain after glucose exhaustion indicates a much slower rate of 6PG formation than consumption and consequently a lower flux through the oxidative PPP. The flux through the oxidative PPP depends on the formation of G6P, which during xylose assimilation can only occur via the non-oxidative PPP and phosphoglucoisomerase (PGI; EC 5.3.1.9) (Figure 1A). The depletion of 6PG in the XI-strain is therefore most likely caused by a reduced flux through the non-oxidative PPP during xylose assimilation.

#### **Significant decrease of hexose phosphates influences metabolite homeostasis in lower glycolysis**

The concentration of the hexose phosphates in glycolysis decreased significantly in both strains during the transition from glucose to xylose fermentation (Figure 5; Additional file 1: Figure S5B). This was a general response in all glycolytic metabolites except for PEP. In bacteria, increased concentration of PEP is a generic response to carbon starvation [51]. Elevated levels of PEP have also been reported in carbon limited or starved yeast cells under aerobic conditions [52-54] but not under anaerobic conditions [37]. In mutant strains it was shown, using traditional biochemical methods, that the hexose phosphates in upper glycolysis play an important role for the activation of the enzymes in lower glycolysis [55,56]. ATP generation and production of ethanol depends on a high activity of pyruvate kinase (PYK; EC 2.7.1.40) which catalyses the last reaction in glycolysis where PEP is converted to pyruvate (PYR) (Figure 1A). This enzyme requires both G6P and F6P for induction [55] and FBP for activation [57] (Figure 1B). FBP is formed from F6P and ATP in the reaction catalysed by 6-phosphofructo kinase (PFK; EC 2.7.1.11) whose activity significantly increases in the presence of fructose 2,6-bisphosphate (F26BP) [58] (Figure 1B). The formation of F26BP is activated through phosphorylation of 6-phosphofructo-2-kinase (PFK26; EC 2.7.1.105) by protein kinase A (PKA) [59] which is most active in the presence of glucose [60] (Figure 1B). This series of metabolic interactions could be the underlying mechanism of the stimulating effect low glucose concentration has on xylose uptake [61-63]. However, it does not explain why xylose is not taken by the engineered yeast cells in a medium with this compound in excess. The accumulation of PEP in the XI-strain suggests a reduced activity of PYK which is consistent with the depletion of FBP. This is most likely connected with the low uptake of xylose by this strain as FBP was not depleted in the XR/XDH-strain (Additional file 1: Figure S5B).

#### **Accumulation of aromatic amino acids is a general response to carbon starvation**

Increased concentrations of alanine (ALA-L), asparagine (ASN-L), glutamate (GLU-L) and the aromatic amino acids has been observed in yeast when starved for carbon under aerobic conditions [52]. Similar results for GLU-L and the aromatic amino acids PHE-L and TRP-L have also been reported for anaerobic conditions [37]. Thus the currently observed accumulation of amino acid pools (Figure 3) corresponds well with previously reported response to carbon starvation, both qualitatively and in many cases also quantitatively (Figure S2D). Aromatic amino acids are the most “expensive” amino acids to synthesize, in terms of ATP requirement [64]. It is therefore unlikely that the observed accumulation is a result of continued synthesis. Instead it is more a reflection of protein degradation with subsequent accumulation of aromatic amino acids, whose aromatic ring is energetically “expensive” or unfavourable to break open. This hypothesis is supported by a measured decrease in total protein content from 0.5 ± 0.05 g protein/g CDW to 0.4 ± 0.03 g protein/g CDW in the XR/XDH-strain when the carbon source changed from glucose to xylose (data not shown).

### **The XR/XDH pathway has no apparent effect on the redox balance**

Declining concentrations of redox cofactors have been observed in cells entering stationary phase [65] and during carbon starvation [37,52]. The introduction of the XR/XDH pathway in *S. cerevisiae* increases the demand for NADPH and the need to re-oxidize NADH (Figure 1A). The increased demand for NADPH is met by increased expression of genes encoding enzymes which catalyse NADPH-yielding reactions, such as those of the oxidative pentose phosphate pathway [24,66]. During xylose assimilation NADPH generation in the oxidative PPP depends on the flux from the non-oxidative PPP via F6P to G6P. The current study shows that the XR/XDH-strain, despite its increased demand for NADPH, is able to maintain a low but constant concentration throughout the xylose phase (Figure S3B). In contrast, the XI-strain is depleted of NADPH already at the beginning of the xylose phase which coincides with the depletion of 6PG after glucose exhaustion (Figure 4D). The depletion of NADPH supports the previous suggestion that the flux through the non-oxidative PPP is low in the XI-strain and it also indicates that this strain suffers from significant carbon limitation.

When the demand for oxidized NADH, i.e. NAD, is not fully met xylitol is secreted as was observed during the transition and xylose phases for the XR/XDH-strain only (Figure 2). The incapacity of both native and recombinant xylose-utilizing XR/XDH yeast strains to reconstitute the NAD/NADH balance under anaerobic conditions has frequently been suggested to be the most important bottleneck in the fermentation of xylose [13,67] and has been the major incentive to develop XI-strains [15]. However, the current targeted metabolite analysis recorded little significant difference between the XR/XDH-strain and the XI-strain in NADH concentration (Figure S3D) and NADH/NAD ratio (Figure 6B). Furthermore, no accumulation of NADH was observed in the XR/XDH-strain during the xylose phase. Although unexpected, it is most likely a result of mixing the cytosolic and mitochondrial pools of redox cofactors which is unavoidable with the extraction method used. If this is the case, it indicates that it is necessary to take into account the compartmentalization of metabolites when evaluating the balancing of redox cofactors in yeast. Another aspect of the redox balance is that several genes associated with respiratory metabolism become derepressed, regardless of oxygenation level, when xylose is the sole carbon source [24,66,68,69]. This specifically leads to increased transcript levels of the three genes coding for malate dehydrogenase (MDH): *MDH1* (mitochondrial), *MDH2* (cytosolic) and *MDH3* (peroxisomal). Under anaerobic conditions Mdh1p converts oxaloacetate to L-malate and is required for reoxidizing the NADH formed during amino acid synthesis inside the mitochondria [70] (Figure 1A). The physiological role of Mdh2p is to generate oxaloacetate for G6P-synthesis during growth on non-fermentable carbon sources and is thus repressed by glucose [71]. The increased expression of both *MDH1* and *MDH2* on xylose gives the cells an enhanced NADH-oxidizing capability. This capability seems to be utilized by the XR/XDH-strain as the concentration of L-malate, the product of the MDH-catalysed reaction, accumulated intracellularly during xylose consumption (Figure 3; Additional file 1: Figure S6D). Even though this path can aid in the balancing of redox cofactors it does not appear to be sufficient as a significant amount of xylitol is still being formed (Figure 2). Instead this path might actually be detrimental for the cells, rather than beneficial, since oxaloacetate is an important precursor metabolite for synthesis of several amino acids, including methionine (Figure 1A). A diversion of flux from these reactions towards L-malate production could result in depletion of these amino acids. Unfortunately we cannot confirm this hypothesis due to lack of measurements of these amino acids and oxaloacetate. However, some support is given by the fact that yeast cells with high Mdh2p-activity only grow half as fast on glucose as the wild-type cells, thus illustrating its negative effect [71].

### **Both yeast strains are able to maintain high energy charge on xylose**

The most commonly used measure to assess the energetic status of cells is the adenylate energy charge (AEC) [72]. It is defined as the ratio between the total adenine nucleotide pool and the sum of the ATP pool and half the ADP pool: (ATP + ½ADP)/(ATP + ADP + AMP). The adenine nucleotides are balanced by adenylate kinase (AK) which catalyses the reaction ATP + AMP ↔ 2 ADP with the ADP pool serving as energy reserve to be used when ATP levels decrease. The AEC has been suggested as an important regulatory signal for controlling the energy balance by directly modulating enzyme activities [73-75]. In *S. cerevisiae* the AEC drops sharply from 0.8 to around 0.5 in response to glucose depletion and continues to fall during prolonged glucose starvation [36,37]. It would therefore have been expected that the low uptake rate of xylose by the two strains (Figure 2) would lead to a decrease in the AEC and ATP concentration, especially in the XI-strain. However, the results show that there is a remarkable consistency between the adenine nucleotide pools in the two strains, clearly demonstrated by the very small confidence intervals in the energy charge (Figure 7A). This implies that the regulatory signal the AEC provides is the same during xylose fermentation as during glucose fermentation. Furthermore, it may reflect an ability of the cells to sense the surplus of external xylose even at very low uptake rates. The observed consistency could thus be the outcome of a mechanism to prevent depletion of ATP which is required to phosphorylate whatever small amount of carbon source is taken up. Therefore the cells do not respond with an energy starvation but only a carbon starvation phenotype. However, the absence of growth despite a high and stable AEC strongly indicates that it is not the required signal for continued growth on xylose.

### **Role of guanine nucleotides in cell division cycle control**

Before a yeast cell commits to start the cell division cycle two criteria need to be fulfilled: accumulation of cell mass to reach a critical size [76] and establishment of a critical rate of protein synthesis [77]. This occurs during the G1 phase of the cycle in which the cell evaluates the nutritional condition of the environment. The guanine nucleotides have been implicated as important regulatory factors in both these processes [78-80]. Rudoni *et al.*[78] reported a value for the GTP/GDP ratio in exponentially growing yeast cells of ca. 4 which is reduced to below one in stationary phase and during glucose starvation. Furthermore, they found a correlation between the cytosolic GTP/GDP ratio and the level of GTP-bound Ras2, which is the active form of this protein. In this form it can bind to adenylate cyclase and trigger the production of cAMP [81] (Figure 1B). The active Ras/cAMP/PKA pathway initiates the biosynthetic machinery by increasing the expression of genes involved in ribosome biogenesis [60] and is thereby required for the cell to reach the size threshold and start the cell cycle [82]. The synthesis of proteins is highly dependent on GTP availability, not only in the elongation step but also in the initiation of translation [83]. The two initiation factors eIF2α and eIF2B have a higher affinity for GDP than GTP and it is therefore not surprising that an imbalance in the GTP/GDP ratio also can affect the rate of protein synthesis [79,80].

The value of the GTP/GDP ratio during exponential growth on glucose obtained in the current study (Figure 7D) agrees well with the results presented by Rudoni *et al.*[78]. The dynamic change in the XI-strain also corresponds with the reported response to carbon exhaustion. The very low GTP/GDP ratio in this strain on xylose could result in low Ras/cAMP activity and rate of protein synthesis leading to an arrest in the G0 phase of the cell cycle. This would support the idea that xylose is not sensed as a fermentable carbon source by the yeast cells [24,66,69] and does not activate the biosynthetic machinery efficiently. However, the GTP/GDP ratio did not change significantly in the XR/XDH-strain during the transition to xylose fermentation, suggesting that the GTP/GDP ratio rather responds to the rate of carbon supply than to what type of carbon source is actually taken up. Hence, other constituents of the nutrient sensing pathways must be involved, e.g. Snf1 and the Tor kinases which can control the initiation of translation by modulating the phosphorylation of eIF2α [84].

### **Other indicators of metabolic status**

In contrast to the GTP/GDP ratio both the guanylate energy charge (GEC) and the GTP/GMP ratio decreased significantly in the two strains when shifting to xylose fermentation (Figure 7B and C). The fast and distinct response in the GEC indicates that this measure could serve as a more sensitive indicator of metabolic status than the AEC. However, yeast lacks an enzyme equivalent to adenylate kinase that can catalyse the conversion of two molecules of GDP to GTP and GMP. Thus, the GEC can be calculated analogous to the AEC, but it is not related to any biological process. Even so, the pools of the adenine and guanine nucleotides are directly interconnected as GTP is used as a phosphate donor in the *de novo* synthesis of ATP and vice versa [85]. This could explain the similar values of the AEC and the GEC (0.82 and 0.86, respectively) during the glucose phase (Figure 7A and B). A 10-fold decrease in the GTP/GMP ratio, similar to the one reported here, as a response to a change from glucose to xylose fermentation has also been measured in a recent study [41]. This highlights the sensitivity of this ratio and could therefore be a better indicator of metabolic status as it is directly related to metabolic processes. However, there have been no studies on how and to what extent the GTP/GMP ratio affects or controls metabolic processes. Even though the role of the guanine nucleotides in yeast metabolism has been less explored than that of the adenine nucleotides, it is clear that the biochemical processes in which these nucleotides participate are required for both growth and survival [86,87]. Recent studies have shown that an imbalance in the guanine nucleotide pool can have detrimental effects on nitrogen metabolism, protein synthesis, viability and proliferation of yeast cells [78,79,88,89]. Still, more systematic investigations of the response in the guanine nucleotide pools under different conditions are needed to understand the role they play in the physiology of yeast.

## **Conclusions**

In this study we determined the time-dependent changes in a selected part of the metabolome of two recombinant strains of *S. cerevisiae*, harbouring either the XR/XDH or the XI pathway, in response to a transition from glucose to xylose fermentation. A fundamental difference between the two strains was the very low uptake rate of xylose by the XI-strain, which gave rise to larger changes in metabolite concentrations than in the XR/XDH-strain. However, even though the XR/XDH-strain had a 10-fold higher uptake rate of xylose it did not continue to grow after glucose depletion. This indicates that factors other than sugar transport limit the growth on xylose. Several distinct responses in the XI-strain were not observed in the XR/XDH-strain: depletion of NADPH and several metabolites in glycolysis, accumulation of PEP and a reduced GTP/GDP ratio. These changes in the metabolome typically direct towards carbon starvation, which appears to be the general response of the XI-strain to xylose. Still, both strains maintained a high and stable energy charge during the transition to xylose, indicating that neither strain is energy limited. In fact, the only response observed in both strains was the accumulation of aromatic amino acids. We suggest that this is related to a general decrease in protein concentration. We also suggest that the redox imbalance between XR and XDH, combined with the poor ability of xylose to repress genes associated with respiratory pathways, give rise to reactions unbeneficial for xylose fermentation.

In light of the stable energy charge, xylose fermentation seems to present a unique condition for *S. cerevisiae* where the energy metabolism is uncoupled from carbon metabolism. The observed discrimination between energy and carbon starvation during xylose fermentation has not been reported previously, but is now made visible through metabolomic analysis of isogenic strains. However, the maintenance of a high energy charge does evidently not correlate with the general metabolic status of the cells; hence, other internal signals could be better indicators. Potential candidates are the guanylate energy charge and the GTP/GMP ratio as they decreased significantly in both strains. The importance of these relationships as indicators of metabolic status merits further investigation as the guanine nucleotides have been shown to play an important regulatory role in cell biology and metabolism. Dynamic metabolomics is a suitable method for exposing such underlying metabolic interactions and to unravel the behaviour of rationally designed cell factories and wild type microorganisms.

## **Methods**

### **Strains and cultivation conditions**

In this study, two recombinant strains of *Saccharomyces cerevisiae*, with the same genetic background but with two different pathways for xylose utilization, were used, i.e. TMB 3057 employing the XR/XDH pathway [21] and TMB 3359 utilizing a xylose isomerase from *Piromyces* sp [22]. Liquid cultures were grown in yeast nitrogen base medium (YNB) without amino acids (1.7 g/L; Difco, Becton, Dickinson and Company, Sparks, MD, USA) with 20 g/L glucose and 5 g/L ammonium sulphate. In anaerobic fermentation, double concentration of YNB (2x YNB) with 20 g/L glucose and 40 g/L xylose was used, supplemented with 5 g/L ammonium sulphate, 400 mg/L Tween 80 and 10 mg/L ergosterol.

### **Batch fermentation**

Cells were pre-grown in YNB medium until late exponential phase and inoculated into the bioreactor at a concentration of 0.04 g CDW/L. Anaerobic fermentation was performed in a 3.1 L Benchtop Fermenter Type KLF2000 bioreactor (Bioengineering AG, Switzerland) with a working volume of 2 litres. The temperature was 30°C, stirring was set at 900 rpm and pH was controlled at 5.5 with 3 M KOH and 1.5 M H_2_SO_4_. Anaerobic conditions were maintained by sparging the culture broth with 0.5 L/min nitrogen gas (Grade 5.0, containing less than 3 ppm oxygen; PanGas AG, Switzerland) controlled by a gas flow controller (Get red-y, Vögtlin Intruments AG, Switzerland). The condenser was connected to a Thermostatic Circulator 2219 (LKB Produkter AB, Sweden) and cooled to 4°C. Foaming was prevented by adding three to four drops of sterile 50% PEG2000. Fermentation experiments were performed in duplicate.

### **Quantification of extracellular substrates and products**

Concentrations of substrates and products in the culture broth were determined by HPLC (Waters, USA) with Aminex HPX-87 H ion exchange column (Bio-Rad, USA) and refractive index detector (RID-6A, Shimadzu, Japan). Mobile phase was 5 mM H_2_SO_4_, the temperature 45°C and the flow rate 0.6 mL/min. Apart from glucose, xylose, xylitol, glycerol and ethanol, no other compounds were detected in the analysis. Stated concentrations of ethanol are not corrected for losses due to evaporation.

### **Biomass determination**

Optical density was measured in triplicate at each time point at 600 nm. Cell dry weight was determined in triplicate by filtering 10 mL of culture through a dried and pre-weighed nitrocellulose filter with a 0.45 μm pore size. The filters were washed with an equal amount of water and dried at 80°C overnight. The filters were allowed to equilibrate to room temperature before weighed again.

### **Quenching, extraction of intracellular metabolites and sample preparation**

The quenching procedure of cell samples was based on the method developed by de Koning *et al.*[25]. Quenching was achieved by quickly adding 1 mL of cell culture to 4 mL of cold, buffered methanol (60% methanol, 10 mM ammonium acetate, pH 7.5, -40°C). The cells were cooled at −40°C in an ethylene glycol bath (FP40-MC, Julabo, Germany) for maximum 30 seconds and then collected by centrifugation at 5000 rpm and −9°C for 5 min. The supernatant was decanted and any residual liquid was removed with a pipette. The pellet was then immediately frozen in liquid nitrogen and stored at −80°C until extraction. Quadruplicate samples were quenched at each time point.

Intracellular metabolites were extracted using the boiling ethanol procedure [90]. Frozen cell pellets were briefly thawed at −40°C before extraction. To extract the metabolites, 1 mL of boiling, buffered ethanol (75% ethanol, 10 mM ammonium acetate, pH 7.5, 85°C) was added to the pellet, directly followed by addition of 100 μL of an internal standard (metabolite extract of yeast grown on ^13^C-labeled glucose) and vortexed quickly. The mixture was incubated 3 min at 85°C with vigorous mixing every 30 seconds. The solution was then shortly cooled back to −40°C and cell debris was spun down by centrifugation at 5000 rpm and −9°C for 3 min. The supernatant was collected in a 1.5 mL tube, frozen in liquid nitrogen and stored at −80°C until further use.

Extracted metabolites were dried by evaporating the solvent at low pressure, 0.01-0.1 mbar, using a Christ Alpha 2–4 LD Plus rotational vacuum concentrator (Adolf Kühner AG, Switzerland) connected to a Chemistry Hybrid Pump RC 6 (Vacuubrand GmbH, Germany) and a Christ AVC 2–33 centrifuge (Adolf Kühner AG, Switzerland). Dried samples were stored at −80°C until further use.

### **Metabolite analysis by LC-MS/MS**

Separation and detection of compounds was achieved on a Waters Acquity UPLC (Waters Corporation, Milford, MA, USA) using a Waters Acquity T3 end-capped reverse phase column with dimensions 150 mm × 2.1 mm × 1.8 mm (Waters Corporation) coupled to a Thermo TSQ Quantum Ultra triple quadrupole mass spectrometer (Thermo Fisher Scientific, Waltham, MA, USA) as previously described [20].

### **Data normalization, calibration and quantification**

Detection and integration of chromatographic peaks was performed using Xcalibur software version 2.0.7 (Thermo Fisher Scientific Inc, USA). The automatic integrations were manually curated to obtain consistent peak boundaries and the results were exported to Matlab R2008a (The MathWorks, USA) where subsequent data processing was performed.

The signal of each analyte was normalized according to the following equation:

(1) $${\tilde{X}}_{\mathrm{ijk}}=X_{\mathrm{ijk}}\frac{\langle Z_{i\cdot }\rangle }{Z_{\mathrm{ijk}}}$$

where *X*_*ijk*_ and ${\tilde{X}}_{\mathrm{ijk}}$are the measured and normalized signals of analyte *i* in sample *k* of time point *j*, respectively, *Z*_*ijk*_ i*s* the signal of the ^13^C-labeled analyte *i* and $\langle \rangle Z_{i\cdot }\rangle \rangle$ is a robust mean of the internal standard signals of analyte *i* in all samples. The robust mean was calculated according to an iterative procedure as outlined in Miller & Miller [91] p. 174].

Seven external standards with known concentrations ranging from 0.14 μM to 100 μM were analyzed together with each batch of samples to be quantified. An initial robust linear regression with a logistic estimator was used to identify possible outliers in the calibration data. Data points with a z-score above three were regarded as outliers and removed from the data set [92]. An ordinary least squares (OLS) regression was then performed with the remaining data points to obtain unbiased model and regression parameters [93]. All calibration curves had at least five data points in the final OLS regression and the adjusted coefficients of determination (adjusted R^2^) were between 0.90 and 1, with the majority around 0.99. A term for the intercept was included in the equation only if the root mean squared error (RMSE) was lower than the RMSE obtained without including the intercept and if it was statistically significant according to a t-test at the 0.05 probability level.

The intracellular concentration corresponding to an estimated sample concentration was calculated from the sample volume and the measured cell dry weight. All intracellular concentrations stated in this paper are mean values of concentrations from duplicate biological experiments and errors are given as a 95% confidence interval of the calculated mean. Samples collected at a particular time point were analyzed for outliers before calculating the mean. This was done by calculating the ratio between |*c*_*ijk*_ – median(*c*_*ij*·_)| and the median absolute deviation (MAD): median(|*c*_*ijk*_ – median(*c*_*ij*·_)|). Measurements with a ratio above four were regarded as outliers and were removed. The total number of replicates at each time point was between four and ten, but the majority of the means were calculated from seven replicates.

### **Chemometrics**

Principal component analysis (PCA) is a statistical method commonly used to analyse data from metabolomics studies [94]. PCA is an unsupervised method used to reduce the dimension of the data by calculating new components which are linear combinations of the original values [95]. The first component is constructed so that it accounts for the highest fraction of the total variance in the data; the second component is constructed to be orthogonal (uncorrelated) to the first component and account for the highest fraction of the variance in this direction. Subsequent components are constructed similarly resulting in a number of components that are fewer than the original number of variables (dimension reduction) and uncorrelated and thus give a unique description of each sample in the new dimension. PCA is classified as an unsupervised method because no prior grouping of the data is required. The result can therefore give an insight into the natural grouping of the data based on the maximisation of the variance criterion. PCA was performed in MATLAB R2010b (Mathworks, USA) using the *princomp* function on quantified concentrations of 37 metabolites from 185 samples. After removing outliers according to the above mentioned criterion the data matrix contained 5.6% missing data points. If only one data point was missing in a certain sampling point and strain it was replaced by the average value of the measured concentrations. If more than one data point was missing, they were replaced by randomly generated numbers from a t-distribution with the number of degrees of freedom equal to the number of samples at that sampling point minus one. The random numbers were converted to concentrations by multiplication with the standard deviation and addition of the mean of the known concentrations. Each metabolite in the imputed data matrix was normalized to the median concentration measured in both strains at the first sampling point and then converted to log_2_ scale.

Hierarchical clustering was performed using the *clustergram* function in MATLAB R2010b with quantified concentrations and normalized chromatographic peak areas of 38 and six metabolites, respectively. The data for the XR/XDH-strain and the XI-strain contained 95 and 90 samples, respectively. Each set of samples was normalized to the corresponding mean concentration or peak area at the first sampling point before converted to log_2_ scale. Euclidean distance was used to calculate the distance between the metabolite profiles and average linkage was used to generate the dendrogram. Data used for the PCA and the hierarchical clustering are given as supplementary information in Additional file 3.

### **Thermodynamic analysis**

The thermodynamic analysis of measured metabolite concentrations in the XR/XDH-strain was performed using anNET version 1.1.06 [23]. Detailed information regarding the calculations and discussion of the results is given in Additional file 2. All the data used in the calculations are given as supplementary information in Additional file 4.

## Metabolites

2PG: 2-phosphoglycerate; 3PG: 3-phosphoglycerate; 6PG: 6- phosphogluconate; 6pgl: 6-phosphogluconolactonate; Acald: Acetaldehyde; ACCOA: Acetyl-Coenzyme A; ADP: Adenosine 5'-diphosphate; AKG: a-ketoglutarate; ALA-L: Alanine; AMP: Adenosine 5'-monophosphate; ARG-L: Arginine; ASN-L: Asparagine; ASP-L: Aspartate; ATP: Adenosine 5'-triphosphate; BPG, Bpg: 1,3-bisphosphoglycerate; cAMP: Cyclic AMP; CIT: Citrate; CoA: Coenzyme A; Cys: Cysteine; DHAP: Dihydroxy acetone phosphate; E4p: Erythrose 4-phosphate; F26BP: Fructose 2,6-bisphosphate; F6P: Fructose 6-phosphate; FAD: Flavin adenine dinucleotide; FBP: Fructose 1,6-bisphosphate; FMN: Riboflavin 5'-monophosphate; FUM: Fumarate; G1P: Glucose 1-phosphate; G6P: Glucose 6-phosphate; GAP, Gap: Glyceraldehyde 3-phosphate; GDP: Guanosine 5'-diphosphate; GLN-L: Glutamine; GLU-L: Glutamate; Gly: Glycine; GLYC3P: Glycerol 3-phosphate; GMP: Guanosine 5'-monophosphate; GTP: Guanosine 5'-triphosphate; GTTox: Oxidized glutathione; GTTred: Reduced glutathione; His: Histidine; ICIT: Isocitrate; Ile: Isoleucine; Leu: Leucine; L-MAL: L-malate; Lys: lysine; Met: Methionine; NAD: Oxidized nicotinamide adenine dinucleotide; NADH: Reduced nicotinamide adenine dinucleotide; NADP: Oxidized nicotinamide adenine dinucleotide phosphate; NADPH: Reduced nicotinamide adenine dinucleotide phosphate; Oaa: Oxaloacetate; PEP: Phoshoenolpyruvate; PHE-L: Phenylalanine; Pro: Proline; PYR, pyr: Pyruvate; R5P: Ribose 5-phosphate; RU5P: Ribulose 5-phosphate; S7P: Sedoheptulose 7-phosphate; Ser: Serine; SUCC: Succinate; Succoa: Succinyl-Coenzyme A; T6P: Trehalose 6-phosphate; Thr: Threonine; TRP-L: Tryptophane; TYR-L: Tyrosine; Val: Valine; xPG: Combined pool of 2PG and 3PG; XU5P: Xylulose 5-phosphate; Xylu: Xylulose;

## Enzymes

AEC, Adenylate energy charge; GEC, Guanylate energy charge; PCA, Principal component analysis; PPP, Pentose phosphate pathway; TCA cycle, Tricarboxylic acid cycle; 2PG, 2-phosphoglycerate; 3PG, 3-phosphoglycerate; 6PG, 6-phosphogluconate; 6pgl, 6-phosphogluconolactonate; Acald, Acetaldehyde; ACCOA, Acetyl-Coenzyme A; ADP, Adenosine 5'-diphosphate; AKG, α-ketoglutarate; ALA-L, Alanine; AMP, Adenosine 5'-monophosphate; ARG-L, Arginine; ASN-L, Asparagine; ASP-L, Aspartate; ATP, Adenosine 5'-triphosphate; BPG, Bpg, 1,3-bisphosphoglycerate; cAMP, Cyclic AMP; CIT, Citrate; CoA, Coenzyme A; Cys, Cysteine; DHAP, Dihydroxy acetone phosphate; E4p, Erythrose 4-phosphate; F26BP, Fructose 2,6-bisphosphate; F6P, Fructose 6-phosphate; FAD, Flavin adenine dinucleotide; FBP, Fructose 1,6-bisphosphate; FMN, Riboflavin 5'-monophosphate; FUM, Fumarate; G1P, Glucose 1-phosphate; G6P, Glucose 6-phosphate; GAP, Gap, Glyceraldehyde 3-phosphate; GDP, Guanosine 5'-diphosphate; GLN-L, Glutamine; GLU-L, Glutamate; Gly, Glycine; GLYC3P, Glycerol 3-phosphate; GMP, Guanosine 5'-monophosphate; GTP, Guanosine 5'-triphosphate; GTTox, Oxidized glutathione; GTTred, Reduced glutathione; His, Histidine; ICIT, Isocitrate; Ile, Isoleucine; Leu, Leucine; L-MAL, L-malate; Lys, lysine; Met, Methionine; NAD, Oxidized nicotinamide adenine dinucleotide; NADH, Reduced nicotinamide adenine dinucleotide; NADP, Oxidized nicotinamide adenine dinucleotide phosphate; NADPH, Reduced nicotinamide adenine dinucleotide phosphate; Oaa, Oxaloacetate; PEP, Phoshoenolpyruvate; PHE-L, Phenylalanine; Pro, Proline; PYR, pyr, Pyruvate; R5P, Ribose 5-phosphate; RU5P, Ribulose 5-phosphate; S7P, Sedoheptulose 7-phosphate; Ser, Serine; SUCC, Succinate; Succoa, Succinyl-Coenzyme A; T6P, Trehalose 6-phosphate; Thr, Threonine; TRP-L, Tryptophane; TYR-L, Tyrosine; Val, Valine; xPG, Combined pool of 2PG and 3PG; XU5P, Xylulose 5-phosphate; Xylu, Xylulose; ACOm, Aconitase EC 4.2.1.3; ACS, Acetyl-CoA synthetase EC 6.2.1.1; ADH, Alcohol dehydrogenase EC 1.1.1.1; AK, Adenylate kinase EC 2.7.4.3; AKGDm, Alpha-ketoglutarate dehydrogenase EC 1.2.4.2; ALDH, Aldehyde dehydrogenase EC 1.2.1.3; CSm, Citrate synthase EC 2.3.3.1; CYR1, Adenylate cyclase EC 4.6.1.1; ENO, Enolase EC 4.2.1.11; FBA, Fructose 1,6-bisphosphate aldolase EC 4.1.2.13; FBP1, Fructose-1,6-bisphosphatase EC 3.1.3.11; FBP26, Fructose-2,6-bisphosphatase EC 3.1.3.46; FRD, FRDm, Fumarate reductase EC 1.3.1.6; FUMASE, FUMASEm, Fumarase EC 4.2.1.2; G6PDH, Glucose-6-phosphate dehydrogenase EC 1.1.1.49; GAPD, Glyceraldehyde-3-phosphate dehydrogenase EC 1.2.1.12; GND, 6-phosphogluconate dehydrogenase EC 1.1.1.44; GPD, NAD-dependent glycerol 3-phosphate dehydrogenase EC 1.1.1.8; GPP, DL-glycerol-3-phosphatase EC 3.1.3.21; HXK, Hexokinase EC 2.7.1.2; IDHm, Isocitrate dehydrogenase EC 1.1.1.41; MDH2, MDH1m, Malate dehydrogenase EC 1.1.1.37; NTH, Trehalase, EC 3.2.1.28; PC, Pyruvate carboxylase EC 6.4.1.1; PDC, Pyruvate decarboxylase EC 4.1.1.1; PDHm, Pyruvate dehydrogenase complex EC 1.2.4.1; PFK, 6-phosphofructo kinase EC 2.7.1.11; PFK26, 6-phosphofructo-2-kinase EC 2.7.1.105; PGAM, Phosphoglycerate mutase EC 5.4.2.1; PGI, Phosphoglucose isomerase EC 5.3.1.9; PGK, 3-phosphoglycerate kinase EC 2.7.2.3; PGL, 6-phosphogluconolactonase EC 3.1.1.31; PKA, cAMP-dependent protein kinase EC 2.7.11.11; PYK, Pyruvate kinase EC 2.7.1.40; RKI, Ribose-5-phosphate ketol-isomerase EC 5.3.1.6; RPE, D-ribulose-5-phosphate 3-epimerase EC 5.1.3.1; SUCOASm, Succinate-CoA ligase EC 6.2.1.5; TAL, Transaldolase EC 2.2.1.2; TKL1, TKL2, Transketolase EC 2.2.1.1; TPI, Triose phosphate isomerase EC 5.3.1.1; TPS1, Trehalose-6-phosphate synthase EC 2.4.1.15; TPS2, Trehalose-6-phosphate phosphatase EC 3.1.3.12; XDH, Xylitol dehydrogenase EC 1.1.1.9; XI, Xylose isomerase EC 5.3.1.5; XR, Xylose reductase EC 1.1.1.307.

## **Competing interests**

The authors declare that BHH is founder and chairman of the board of C5 Ligno Technologies in Lund AB, Sweden.

## **Authors' contributions**

BB conceived the study, carried out the experimental work, the analysis and interpretation of the data and drafted the manuscript. DH participated in the design of the study and in the experimental work. BBH and EWJN participated in the design of the study, contributed to the interpretation of the data and critically revised the manuscript. US contributed by critically revising the content of the manuscript. All authors’ read and approved the final manuscript.

## Supplementary Material

Additional file 1 Supplementary figures and tables. Click here for file

Additional file 2 **Thermodynamic evaluation of metabolite concentrations [**[20,24-35]**].** Click here for file

Additional file 3 Data used for PCA and cluster analysis. Click here for file

Additional file 4 Data used for validation of metabolite concentrations. Click here for file
