# Supplementary material for: Dynamic metabolomics differentiates between carbon and energy starvation in recombinant Saccharomyces cerevisiae fermenting xylose
Source: Biotechnol Biofuels. 2012 May 15;5:34. doi: 10.1186/1754-6834-5-34 (PMC3462113; doi:10.1186/1754-6834-5-34)
Supplement: Additional file 1 — Supplementary figures and tables. [file 1754-6834-5-34-S1.pdf]

Additional file 1

Figures

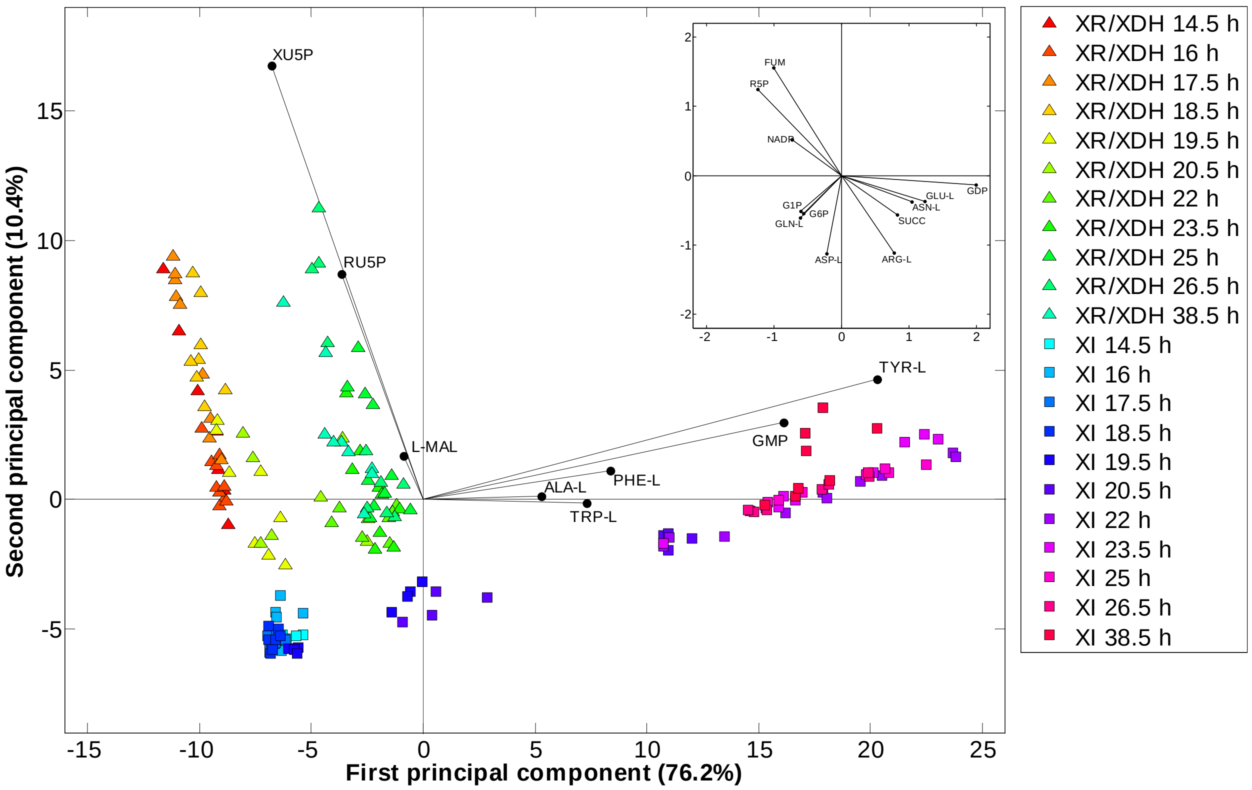

Figure S1 – Global analysis of the data by PCA.

Scores of the data points in the first two dimensions of the PCA analysis and the loadings of the 20 most influential metabolites. The first component accounted for 76.2% of the variance and separated the data by sampling time. The second component accounted for 10.4% of the variance and separated the data by strain background. Three distinct phases were identified during the fermentation: a glucose phase (14.5 h to 18.5 h), a transition phase (18.5 h to 22 h) and a xylose phase (22 h to 38.5 h). The two strains cluster closer together during the glucose phase as compared with the xylose phase. The metabolite loadings were scaled up 30 times in PC1 and 20 times in PC2 to increase readability.

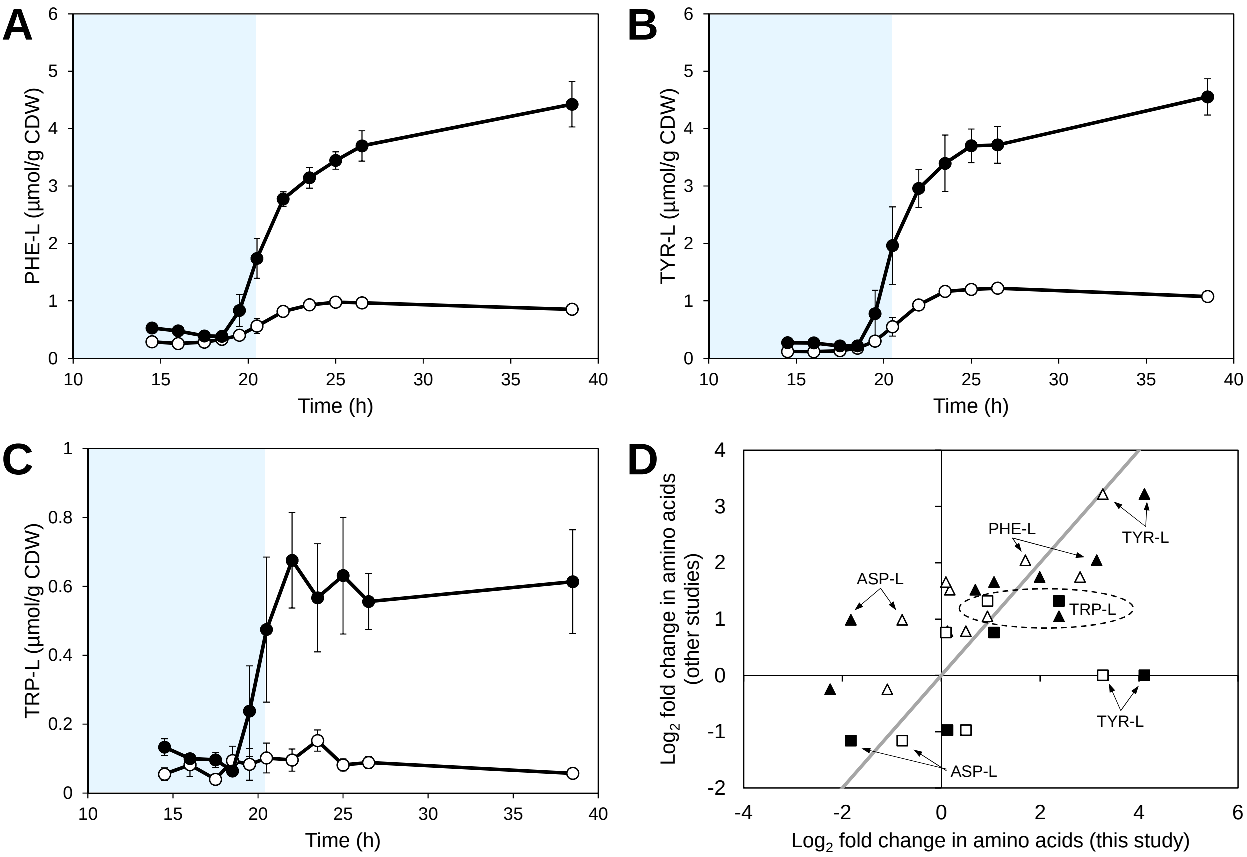

Figure S2 – Accumulation of aromatic amino acids.
Intracellular concentrations of **A**) L-phenylalanine (PHE-L), **B**) L-tyrosine (TYR-L) and **C**) L-tryptophan (TRP-L) during anaerobic batch fermentation of 20 g/L glucose and 40 g/L xylose using strains TMB3057 (XR/XDH, white markers) and TMB3359 (XI, black markers). Errors are given as 95% confidence intervals of the means calculated from duplicate experiments for each strain ($6 \leq n \leq 10$). The shaded area indicates the period until glucose depletion. **D**) Comparison of $\log_2$ fold change in the concentrations of amino acids between this study and Brauer *et al*. [47] (triangles) and Klimacek *et al*. [27] (squares).

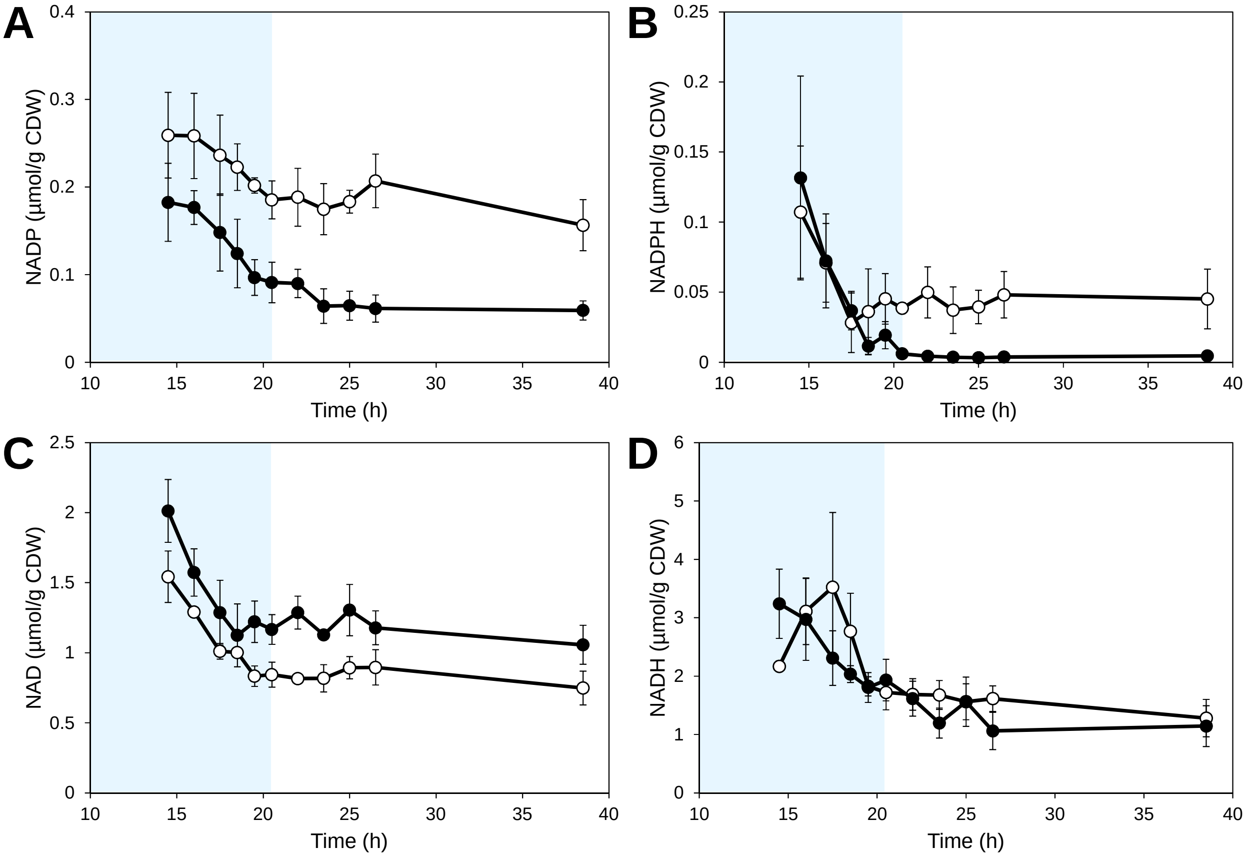

Figure S3 – Dynamics in redox cofactors

Intracellular concentrations of **A**) NADP, **B**) NADPH, **C**) NAD and **D**) NADH during anaerobic batch fermentation of 20 g/L glucose and 40 g/L xylose using strains TMB3057 (XR/XDH, white markers) and TMB3359 (XI, black markers). Errors are given as 95% confidence intervals of the means calculated from duplicate experiments for each strain ($4 \leq n \leq 10$). The shaded area indicates the period until glucose depletion.

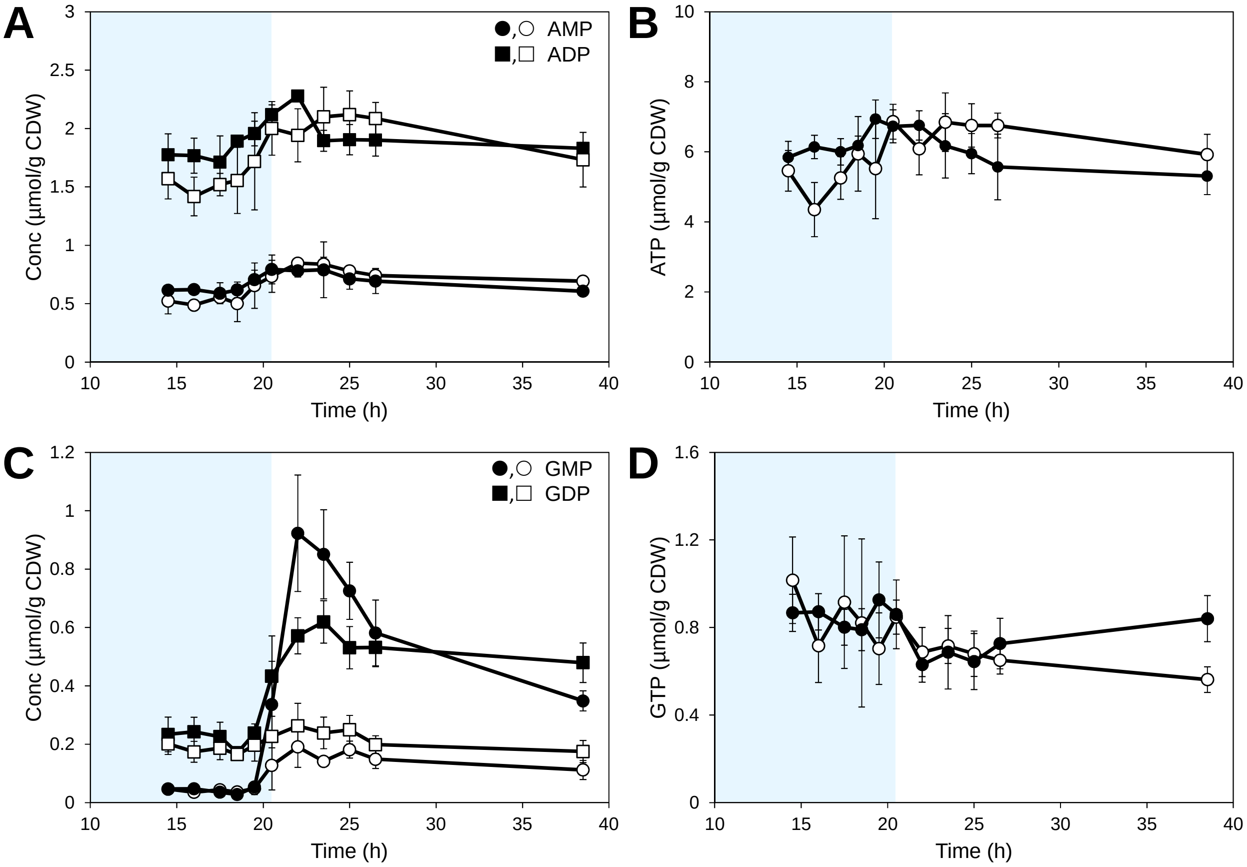

Figure S4 – Dynamics in the purine nucleotide pools

Intracellular concentrations of **A**) AMP and ADP, **B**) ATP, **C**) GMP and GDP and **D**) GTP during anaerobic batch fermentation of 20 g/L glucose and 40 g/L xylose using strains TMB3057 (XR/XDH, white markers) and TMB3359 (XI, black markers). Errors are given as 95% confidence intervals of the means calculated from duplicate experiments for each strain ($5 \leq n \leq 11$). The shaded area indicates the period until glucose depletion.

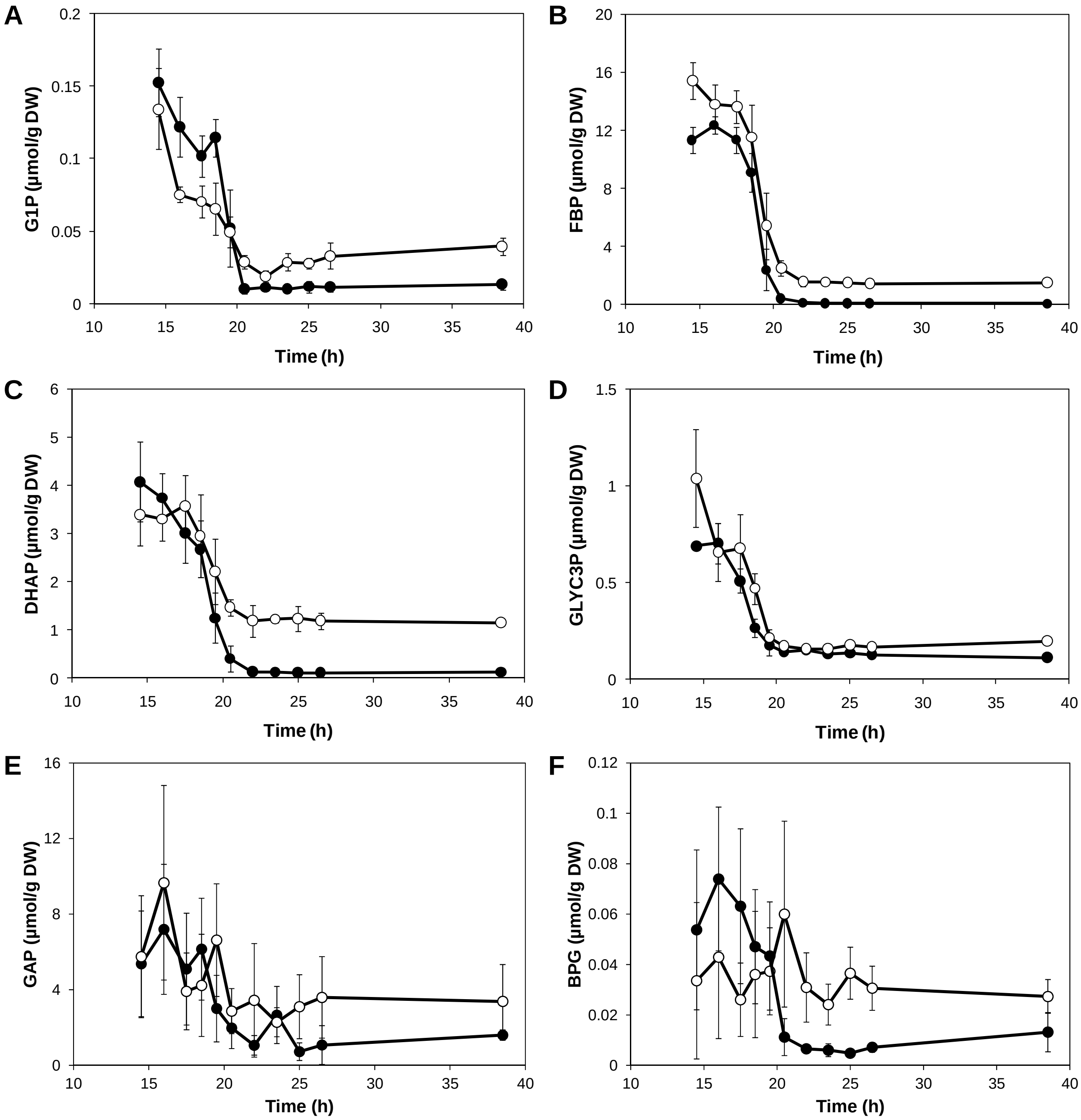

Figure S5 – Additional metabolites in glycolysis.

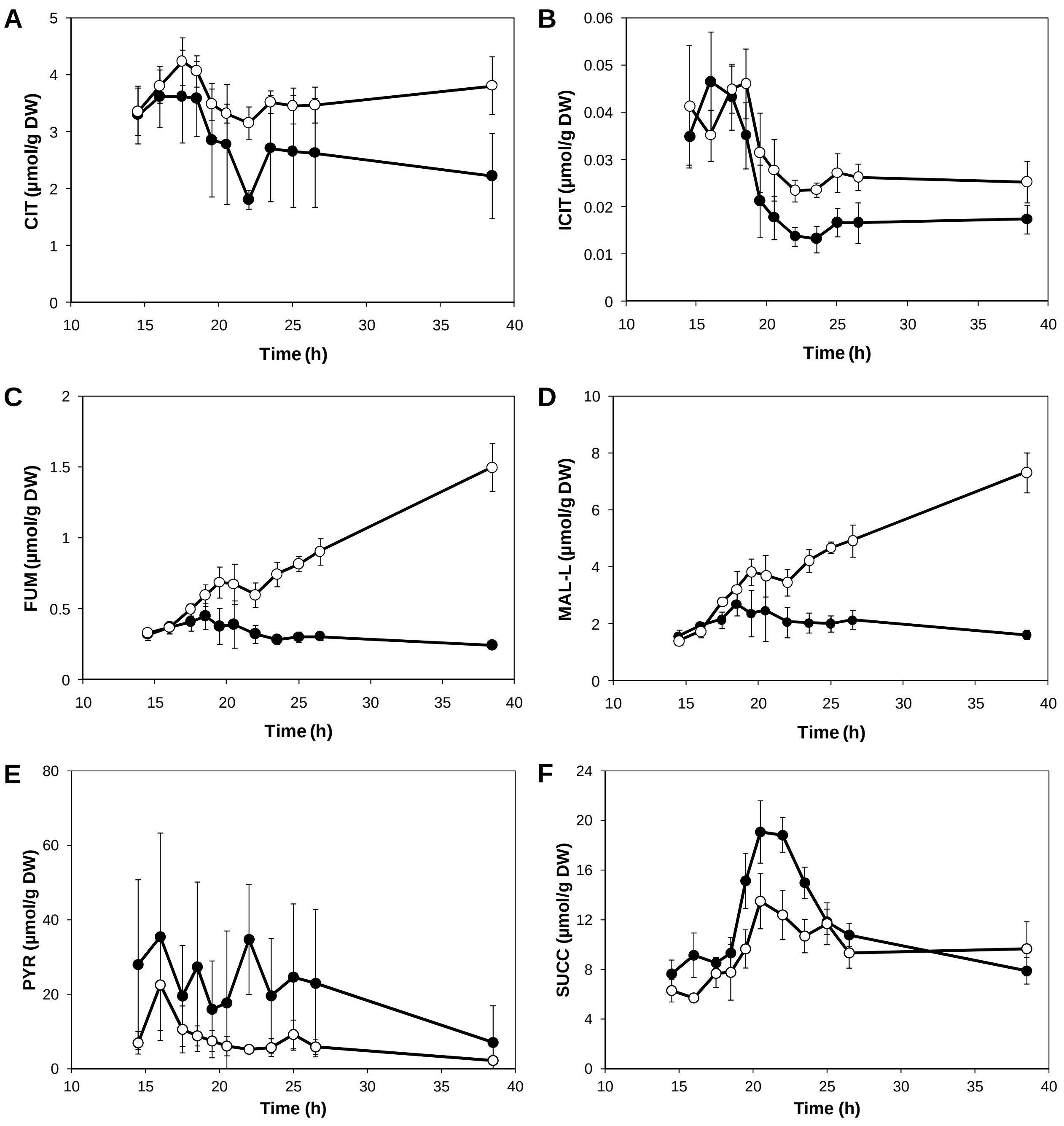

Figure S6 – Additional metabolites in the TCA-cycle.

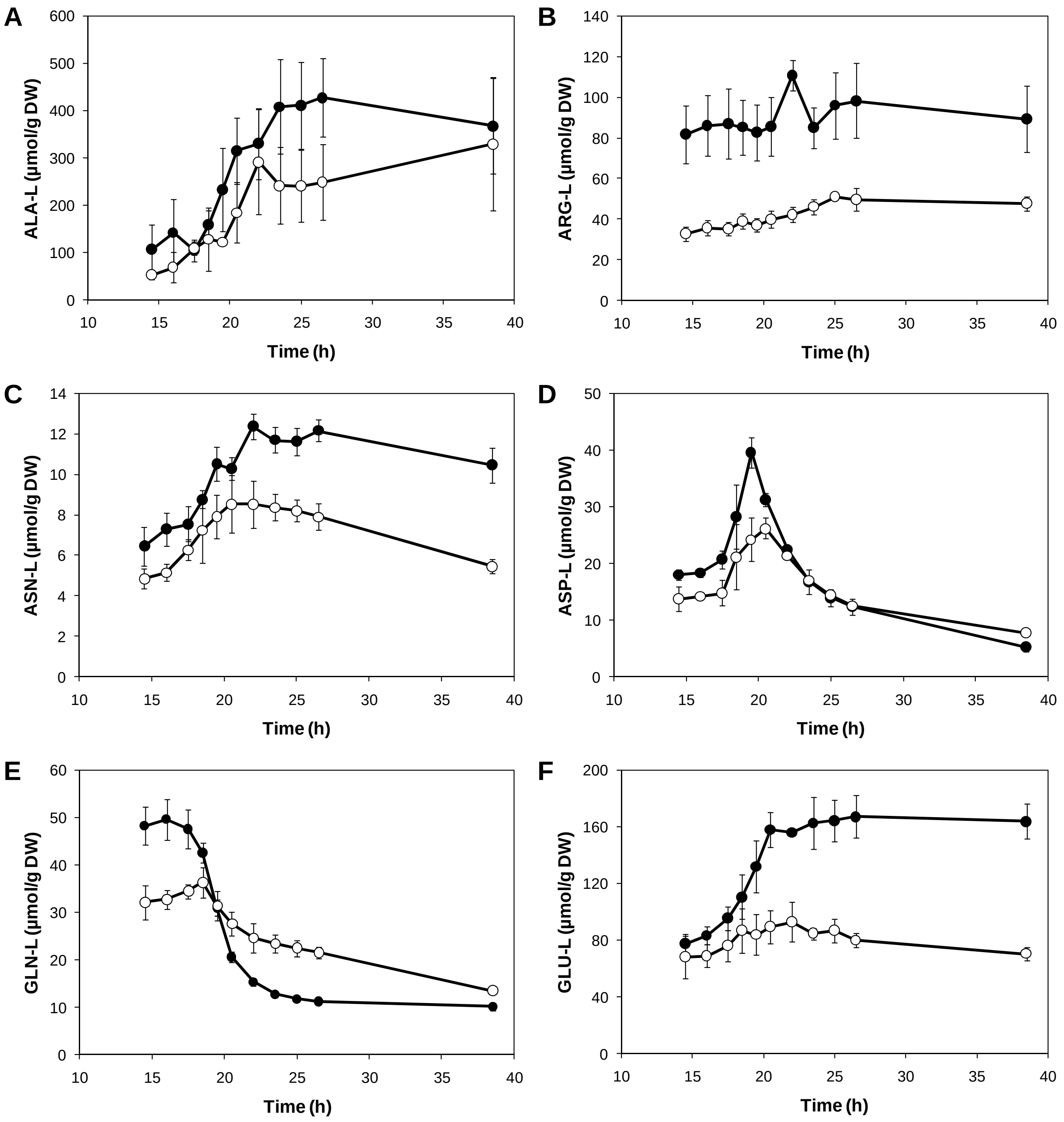

Figure S7 – Additional amino acids.

Tables

Table S1 – Product yields on substrate during anaerobic batch fermentation. Fermentation was performed with the strains TMB 3057 (XR/XDH) and TMB 3359 (XI) using 2X YNB with 20 g L^{-1} glucose and 50 g L^{-1} xylose as substrates.

Yield (g/g)	Mixed sugar		Xylose only		Total sugar	
	TMB 3057	TMB 3359	TMB 3057	TMB 3359	TMB 3057	TMB 3359
Y_{sx}	0.07±0.004	0.08±0.03	0.02±0.003	0.07±0.05	0.05±0.005	0.09±0.02
Y_{se}	0.28±0.03	0.28±0.13	0.13±0.02	-0.06±0.36	0.23±0.02	0.31±0.08
Y_{sg}	0.13±0.01	0.07±0.03	0.03±0.01	0.08±0.17	0.08±0.01	0.08±0.02
Y_{sxylt}	0.09±0.03	0.002±0.003	0.64±0.03	0.17±0.06	0.34±0.06	0.011±0.006

Yields: Y_{sx}, biomass yield on substrate; Y_{se}, ethanol yield on substrate; Y_{sg}, glycerol yield on substrate; Y_{sxylt}, xylitol yield on substrate.

Table S2. Metabolites with significantly different means between the XR/XDH-strain and the XI-strain during anaerobic batch fermentation. Two-sided t-tests were systematically performed for each metabolite and at each sampling point, to determine if there were any significant differences between the two strains. The tests were performed at the 0.05 significance level, assuming equal variance in the two populations. (* $p < 0.01$; ** $p < 0.005$; *** $p < 0.001$)

Time (h)	Number of metabolites	Metabolites			
		**Sugar phosphates**	**Nucleotides and cofactors**	**Amino acids**	**TCA-cycle intermediates**
14.5	19	G6P(***), R5P(***), GLYC3P(**), Xu5P(**), Ru5P(***), 6PG(***), xPG(***), FBP(***)	NAD(**), NADH, NADP	ARG-L(***), ASN-L(**), GLN-L(***), TYR-L(***), PHE-L(***), ASP-L(***), TRP-L(***)	SUCC
16	23	G6P(***), R5P(***), G1P(***), Xu5P(***), Ru5P(***), 6PG(***)	GMP(**), NAD(**), AMP(***), GDP, NADP(*), ADP(**), ATP(***)	ARG-L(***), ALA-L, ASN-L(***), GLN-L(***), TYR-L(***), GLU-L(*), PHE-L(***), ASP-L(***)	SUCC(***), ICIT
17.5	20	G6P(***), R5P(***), G1P(**), Xu5P(***), Ru5P(***), FBP(**)	GMP, NAD, NADP(*), ATP	ARG-L(***), ASN-L(*), GLN-L(***), TYR-L(***), GLU-L(*), PHE-L(***), ASP-L(***), TRP-L(***)	L-MAL(***), FUM
18.5	16	G6P(***), R5P(***), GLYC3P(***), G1P(***), Xu5P(***), Ru5P(***), xPG, FBP	NADH, NADP(***)	ARG-L(***), GLN-L(**), GLU-L, PHE-L	FUM, ICIT
19.5	22	R5P(***), Xu5P(***), Ru5P(*), DHAP, 6PG(***), xPG(***), FBP	NAD(***), NADP(***), GTP, NADPH(**)	ARG-L(***), ALA-L, ASN-L(***), TYR-L, GLU-L(***), PHE-L(**), ASP-L(***), TRP-L	SUCC(***), L-MAL(**), FUM(***)
20.5	30	G6P(***), R5P(**), F6P(**), GLYC3P, G1P(***), Xu5P(***), Ru5P(***), DHAP(***), 6PG(***), xPG, PEP(**), FBP(***)	GMP, NAD(***), GDP, NADP(***), NADPH(***)	ARG-L(***), ALA-L(*), ASN-L, GLN-L(***), TYR-L(***), GLU-L(***), PHE-L(***), ASP-L(***), TRP-L(**)	SUCC(**), L-MAL, FUM(*), ICIT
22	31	G6P(***), R5P(*), F6P(***), G1P(***), Xu5P(***), Ru5P(***), DHAP(***), 6PG(***), xPG(***), PEP(***), FBP(***)	GMP(***), NAD(***), GDP(***), NADP(***), ADP(**), NADPH(***)	ARG-L(***), ASN-L(***), GLN-L(***), TYR-L(***), GLU-L(***), PHE-L(***), ASP-L, TRP-L(***)	PYR(**), SUCC(***), L-MAL(***), FUM(***), CIT(***), ICIT(***)
23.5	30	G6P(***), R5P(***), F6P(***), GLYC3P(***), G1P(***), Xu5P(***), Ru5P(**), DHAP(***), 6PG(***), xPG(***), PEP, FBP(***)	GMP(***), NAD(***), GDP(***), NADH(*), NADP(***), NADPH(***)	ARG-L(***), ALA-L(*), ASN-L(***), GLN-L(***), TYR-L(***), GLU-L(***), PHE-L(***), TRP-L(***)	SUCC(***), L-MAL(***), FUM(***), ICIT(***)
25	29	G6P(***), R5P(***), F6P(***), GLYC3P(**), G1P(***), Xu5P(***), Ru5P(***), DHAP(***), 6PG(***), xPG(***), PEP(**), FBP(***)	GMP(***), NAD(***), GDP(***), NADP(***), NADPH(***)	ARG-L(***), ALA-L(**), ASN-L(***), GLN-L(***), TYR-L(***), GLU-L(***), PHE-L(***), TRP-L(***)	L-MAL(***), AKG(**), FUM(***), ICIT(***)
26.5	33	G6P(***), R5P(***), F6P(***), GLYC3P(***), G1P(***), Xu5P(**), Ru5P(**), DHAP(***), 6PG(***), xPG(***), PEP, FBP(***)	GMP(***), NAD(**), GDP(***), NADH(**), NADP(***), ADP, NADPH(***), ATP(*)	ARG-L(***), ALA-L(**), ASN-L(***), GLN-L(***), TYR-L(***), GLU-L(***), PHE-L(***), TRP-L(***)	SUCC, L-MAL(***), FUM(***), CIT, ICIT(***)
38.5	31	G6P(***), R5P(***), F6P(***), GLYC3P(***), G1P(***), Xu5P(***), Ru5P, DHAP(***), 6PG(***), xPG(***), PEP, FBP(***)	GMP(***), NAD(**), AMP(**), GDP(***), NADP(***), GTP(***), NADPH(**)	ARG-L(***), ASN-L(***), GLN-L(***), TYR-L(***), GLU-L(***), PHE-L(***), ASP-L(***), TRP-L(***)	L-MAL(***), FUM(***), CIT(***), ICIT(**)
