# Supplementary material for: Dynamic metabolomics differentiates between carbon and energy starvation in recombinant Saccharomyces cerevisiae fermenting xylose
Source: Biotechnol Biofuels. 2012 May 15;5:34. doi: 10.1186/1754-6834-5-34 (PMC3462113; doi:10.1186/1754-6834-5-34)
Supplement: Additional file 2 — Thermodynamic evaluation of metabolite concentrations [[20,24-35]]. [file 1754-6834-5-34-S3.pdf]

Additional file 3

Thermodynamic evaluation of metabolite concentrations

The thermodynamic analysis of measured metabolite concentrations in the XR/XDH-strain was performed using anNET version 1.1.06 [1]. anNET can evaluate the feasibility of a reaction system constrained by flux directions and ranges of metabolite concentrations. When the system is feasible anNET can calculate the maximum and minimum values of feasible metabolite concentrations.

Calculation of flux distributions

We used the genome scale model iMM904 of *S. cerevisiae* [2] as the reaction network. The reaction network was slightly modified to represent the recombinant XR/XDH-strain: i) the reactions associated with the *GRE*3 gene were deleted except for the NADPH-dependent xylose reductase reaction, ii) a NADH-dependent xylose reductase reaction was added to the model and iii) the xylitol dehydrogenase reaction was made reversible. The glukokinase reaction (GLUK) and the two irreversible alcohol deydrogenase reactions (ALCD2if and ALCD2ir) were also deleted to avoid loops in the flux variability analysis.

The COBRA Toolbox [3] was used to calculate a set of flux directions in the XR/XDH-strain under two conditions. Data from anaerobic chemostat experiments at dilution rate 0.06 h^{-1} with 10 g/L glucose and 10 g/L xylose in the feed [4] was used to simulate the fermentation of a mixed sugar solution. In the case where xylose was the sole carbon source, data from anaerobic batch cultivation of a xylose-growing strain [5] was used to calculate the flux distribution. In both conditions the oxygen uptake rate was set to zero to simulate anaerobic conditions and the maximum uptake rates of sterols, fatty acids, vitamins and trace elements were specified according to Snitkin *et al.* [6]. The objective function was maximisation of biomass formation in both cases. A flux variability analysis was performed to obtain a range of flux directions for each reaction and to identify reactions which never held a flux. Reactions with an absolute flux below 10^{-6} were also regarded as zero and removed from the reaction network. The reduced model consisted of 722 reactions and 590 metabolites.

Input data

To convert measured metabolite concentrations from micro moles per gram cell dry weight (µmol/g CDW) to moles per litre a factor of 1.5 µL cytosolic volume per mg cell dry weight was used [7]. Two measured concentration ranges were not included in the thermodynamic calculations: that of 1,3-bisphosphoglycerate (BPG) which was associated with rather large errors (Fig. S5, Additional file 1) and that of glucose 1-phosphate (G1P) since it only participates in one reaction that could be evaluated. Hence, the concentrations of G1P and BPG were intentionally allowed to vary within the default range (1×10^{-4}-10 mM) in the evaluation. In this study we used the extended set of Gibbs energies of formation supplied with the anNET toolbox for several common biological compounds and complemented these with estimated data for L-arginine, L-ornithine, N(omega)-(L-Arginino)succinate, L-citrulline, acetoin and sedoheptulose-1,7-bisphosphate using the group contribution method [8]. All the data used in the calculations are given as supplementary information in Additional file 4.

Results

The feasibility was evaluated at each sampling point and the measured concentrations of BPG were found to be infeasible in the first four sampling points while all but one measured concentration of G1P fell within the calculated ranges (Table 1). In addition, the measured concentration of glyceraldehyde 3-phosphate (GAP) was found to be thermodynamically infeasible in all time points. The minimum measured concentration of GAP was between 3 and 14 times higher than the maximum calculated concentration (Table 1). Inconsistent concentrations of GAP have previously been identified [1] but the magnitude of the currently obtained concentrations (mM scale) is significantly higher than earlier reported (µM scale) [9-12]. The discrepancy could be due to a combination of the extraction method applied and limitations in the LC-MS/MS method, which yields broad and indistinct chromatographic peaks of GAP with low intensities [13]. This led to inconsistent normalization of GAP peak areas to the internal standard and poor calibration curves for GAP, possibly due to degradation of this compound during the extraction procedure. Apart from BPG, G1P and GAP, all other metabolites evaluated were within the feasible ranges in all 11 time points.

Table 1. Feasibility of measured concentrations of GAP, BPG and G1P. Measured concentration ranges of glyceraldehyde 3-phosphate (GAP), 1,3-bisphospho-D-glycerate (BPG) and glucose 1-phosphate (G1P) based on the 95% confidence intervals compared with thermodynamically feasible ranges calculated using the anNET toolbox.

Time (h)	GAP (mM)		BPG (mM)[a]		G1P (mM)[a]	
	Measured	Calculated	Measured	Calculated	Measured	Calculated
14.5	1.7–6.0	0.005–0.26	0.0017–0.043	0.098–5.5	0.13–0.18	1×10^{-4}–0.18
16	3.0–9.9	0.007–0.25	0.0071–0.050	0.075–2.5	0.11–0.14	1×10^{-4}–0.13
17.5	1.3–4.0	0.010–0.24	0.0077–0.027	0.065–1.7	0.087–0.12	1×10^{-4}–0.095
18.5	1.0–4.6	0.013–0.23	0.0073–0.041	0.045–0.81	0.11–0.13	1×10^{-4}–0.076
19.5	2.4–6.4	0.014–0.18	0.013–0.036	0.023–0.29	0.026–0.079	1×10^{-4}–0.052
20.5	1.1–2.7	0.028–0.11	0.015–0.065	0.027–0.11	0.007–0.013	1×10^{-4}–0.028
22	0.29–4.3	0.028–0.086	0.011–0.030	0.017–0.053	0.009–0.012	1×10^{-4}–0.016
23.5	1.0–2.0	0.042–0.083	0.011–0.021	0.020–0.038	0.008–0.012	1×10^{-4}–0.019
25	0.94–3.2	0.050–0.080	0.018–0.031	0.019–0.031	0.008–0.016	1×10^{-4}–0.022
26.5	0.96–3.8	0.057–0.078	0.015–0.026	0.022–0.030	0.008–0.014	1×10^{-4}–0.026
38.5	0.95–3.6	0.074–0.079	0.014–0.023	0.017–0.019	0.01–0.017	1×10^{-4}–0.035

References

1. Zamboni N, Kummel A, Heinemann M: **anNET: A tool for network-embedded thermodynamic analysis of quantitative metabolome data.** *BMC Bioinformatics* 2008, **9**.
2. Mo M, Palsson B, Herrgard M: **Connecting extracellular metabolomic measurements to intracellular flux states in yeast.** *BMC Syst Biol* 2009, **3:**37.
3. Becker SA, Feist AM, Mo ML, Hannum G, Palsson BO, Herrgard MJ: **Quantitative prediction of cellular metabolism with constraint-based models: the COBRA Toolbox.** *Nat Protocols* 2007, **2:**727-738.
4. Wahlbom CF, Eliasson A, Hahn-Hägerdal B: **Intracellular fluxes in a recombinant xylose-utilizing *Saccharomyces cerevisiae* cultivated anaerobically at different dilution rates and feed concentrations.** *Biotechnol Bioeng* 2001, **72:**289-296.
5. Runquist D, Hahn-Hagerdal B, Bettiga M: **Increased expression of the oxidative pentose phosphate pathway and gluconeogenesis in anaerobically growing xylose-utilizing *Saccharomyces cerevisiae*.** *Microb Cell Fact* 2009, **8**.
6. Snitkin E, Dudley A, Janse D, Wong K, Church G, Segre D: **Model-driven analysis of experimentally determined growth phenotypes for 465 yeast gene deletion mutants under 16 different conditions.** *Genome Biol* 2008, **9:**R140.
7. de Koning W, van Dam K: **A method for the determination of changes of glycolytic metabolites in yeast on a subsecond time scale using extraction at neutral pH.** *Anal Biochem* 1992, **204:**118-123.
8. Jankowski MD, Henry CS, Broadbelt LJ, Hatzimanikatis V: **Group contribution method for thermodynamic analysis of complex metabolic networks.** *Biophys J* 2008, **95:**1487-1499.
9. Klimacek M, Krahulec S, Sauer U, Nidetzky B: **Limitations in xylose-fermenting *Saccharomyces cerevisiae*, made evident through comprehensive metabolite profiling and thermodynamic analysis.** *Appl Environ Microbiol* 2010, **76:**7566-7574.
10. Cipollina C, ten Pierick A, Canelas AB, Seifar RM, van Maris AJA, van Dam JC, Heijnen JJ: **A comprehensive method for the quantification of the non-oxidative pentose phosphate pathway intermediates in *Saccharomyces cerevisiae* by GC-IDMS.** *J Chromatogr B* 2009, **877:**3231-3236.
11. Hynne F, Dano S, Sorensen PG: **Full-scale model of glycolysis in *Saccharomyces cerevisiae*.** *Biophys Chem* 2001, **94:**121-163.
12. Altintas MM, Eddy CK, Zhang M, McMillan JD, Kompala DS: **Kinetic modeling to optimize pentose fermentation in *Zymomonas mobilis*.** *Biotechnol Bioeng* 2006, **94:**273-295.
13. Buescher JM, Moco S, Sauer U, Zamboni N: **Ultrahigh performance liquid chromatography-tandem mass spectrometry method for fast and robust quantification of anionic and aromatic metabolites.** *Anal Chem* 2010, **82:**4403-4412.
